# Large‐scale genomic sequencing reveals adaptive opportunity of targeting mutated‐PI3Kα in early and advanced HER2‐positive breast cancer

**DOI:** 10.1002/ctm2.589

**Published:** 2021-11-04

**Authors:** Lin‐Wei Guo, Xiao‐Guang Li, Yun‐Song Yang, Xun‐Xi Lu, Xiang‐Chen Han, Guan‐Tian Lang, Li Chen, Zhi‐Ming Shao, Xin Hu

**Affiliations:** ^1^ Precision Cancer Medicine Center Department of Breast Surgery Key Laboratory of Breast Cancer in Shanghai Fudan University Shanghai Cancer Center Shanghai P.R. China

**Keywords:** early and advanced HER2‐positive breast cancer, library screening, prospective sequencing, shifty PI3Kα treatment strategy

## Abstract

**Background:**

Few studies have discussed the contradictory roles of mutated‐PI3Kα in HER2‐positive (HER2+) breast cancer. Thus, we characterised the adaptive roles of PI3Kα mutations among HER2+ tumour progression.

**Methods:**

We conducted prospective clinical sequencing of 1923 Chinese breast cancer patients and illustrated the clinical significance of *PIK3CA* mutations in locally advanced and advanced HER2+ cohort. A high‐throughput *PIK3CA* mutations‐barcoding screen was performed to reveal impactful mutation sites in tumour growth and drug responses.

**Results:**

*PIK3CA* mutations acted as a protective factor in treatment‐naïve patients; however, advanced/locally advanced patients harbouring mutated‐PI3Kα exhibited a higher progressive disease rate (100% vs. 15%, *p = *.000053) and a lower objective response rate (81.7% vs. 95.4%, *p *= .0008) in response to trastuzumab‐based therapy. Meanwhile, patients exhibiting anti‐HER2 resistance had a relatively high variant allele fraction (VAF) of *PIK3CA* mutations; we defined the VAF > 12.23% as a predictor of poor anti‐HER2 neoadjuvant treatment efficacy. Pooled mutations screen revealed that specific PI3Kα mutation alleles mediated own biological effects. *PIK3CA* functional mutations suppressed the growth of HER2+ cells, but conferred anti‐HER2 resistance, which can be reversed by the PI3Kα‐specific inhibitor BYL719.

**Conclusions:**

We proposed adaptive treatment strategies that the mutated *PIK3CA* and amplified *ERBB2* should be concomitantly inhibited when exposing to continuous anti‐HER2 therapy, while the combination of anti‐HER2 and anti‐PI3Kα treatment was not essential for anti‐HER2 treatment‐naïve patients. These findings improve the understanding of genomics‐guided treatment in the different progressions of HER2+ breast cancer.

## Graphical abstract

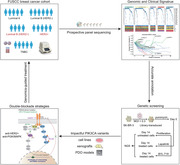

## 1 INTRODUCTION

Breast cancer harbouring gene amplification of epidermal growth factor receptor 2 (*ERBB2*, which encodes HER2) or overexpression of HER2 is categorised as HER2+ breast cancer, which occurs in approximately 15%–20% of all breast cancer cases.^1^ Half of the HER2+ breast cancer patients are initially nonresponsive or acquire resistance to anti‐HER2 therapy,^2^, ^3^ and oncogenic mutations of *PIK3CA* play a vital role in constitutively activating the phosphoinositide 3‐kinase (PI3K)/protein kinase B (AKT)/mechanistic target of rapamycin (mTOR) downstream signalling pathway.^4^

About 20%–50% of breast cancers harbour *PIK3CA* mutations, of which 35% are hormone receptor positive (HR+) and 25% are HER2+ subtypes.^5^, ^6^ Clinical trials, including GeparQuattro,^7^ GeparQuinto,^8^ GeparSixto,^9^ NeoALTTO^10^ and CHERLOB,^11^ all demonstrated that tumours harbouring a *PIK3CA* mutation were significantly associated with a lower pathological complete response (pCR) rate than those with wild‐type *PIK3CA* in the neoadjuvant cohort^12^, ^13^ and that the mutations were related to poor treatment efficacy in advanced breast cancer.^14^, ^15^ Fortunately, the advent of the PI3K inhibitor solved the dilemma. Phase II trial, NeoPHOEBE,^16^ demonstrated the combination of trastuzumab, paclitaxel and neoadjuvant pan‐PI3K inhibitor was ineffective, mainly due to the severe adverse effect of buparlisib; however, the ER+HER2+ subgroup showed a higher ORR tendency and Ki67 reduction, indicating a promising future of PI3K‐targeted therapy and then better‐tolerated subunit‐specific second‐generation PI3K inhibitors occurred. SOLAR‐1 trial^17^ showed great improvements in prolonging progression‐free survival (PFS) and overall survival (OS) with the addition of PI3Kα‐specific inhibitor BYL719 in mutated‐PI3Kα, HR+, HER2‐negative (HER2–) advanced breast cancer who have received endocrine therapy previously. Meanwhile, Loibl et al. reported that no definite conclusions could be drawn regarding survival of DFS or OS between the cohorts with *PIK3CA* wild‐type versus mutant tumours in GeparQuattro, GeparQuinto and GeparSixto studies.^7^, ^8^, ^9^ Given the enormous patients with amplified‐*ERBB2* and mutated‐*PIK3CA*, here we conducted a large‐scale prospective sequencing to investigate the associations between *PIK3CA* mutations and anti‐HER2 therapy.

The OPPORTUNE trial^18^ found that patients with kinase domain mutations (H1047R) responded well to anastrozole alone and showed no benefit from the addition of PI3Kα/δ inhibitor pictilisib, while patients with helical domain (E545K, E542K) showing a particularly poor response to anastrozole alone which could be reversed by the addition of pictilisib ; one of the possibilities was due to the different activating levels of PI3K signalling caused by distinct mutation sites in *PIK3CA*.^19^, ^20^ So far, the existing research mainly focused on the specific hotspot mutations in HR+HER2‐ breast cancer, the functional characteristics and clinical significance of rare mutations in the PI3K of HER2+ breast cancer subtype were worthy investigated.

In this study, we conducted a large‐scale prospective study using the Fudan breast cancer‐specific NGS panel, summarising the correlation between clinical and genomic variation in RTK‐PI3K‐MAPK pathways, and illustrated the adaptive roles of *PIK3CA* mutations in HER2+ tumour progression. Using pooled *PIK3CA* mutation screens, we revealed the contributions of various *PIK3CA* alleles to tumour growth and drug responses, providing the evolutionary therapeutics to overcome anti‐HER2 resistance.

## 2 METHODS

### 2.1 Fudan University Shanghai Cancer Center (FUSCC) breast cancer prospective sequencing cohort

A total of 1923 Chinese patients diagnosed with breast cancer from April 1, 2018 to September 31, 2019 at the Department of Breast Surgery at FUSCC were prospectively recruited to the FUSCC BC sequencing cohort.^21^ We utilised a custom‐designed panel of 484 breast cancer‐associated hypermutated genes based on the integration of The Cancer Genome Atlas (TCGA),^5^ Memorial Sloan Kettering Cancer Center (MSKCC)^22^ and FUSCC‐TNBC datasets^23^ and contained targetable biomarkers approved for clinical use or those used in experimental therapies (Table S1). Genomic DNA (gDNA) from treatment‐naive tumour samples and matched blood samples was extracted, sheared through Covaris M220 instrument, followed by end‐repair, polyA‐tailing and adapter ligation instructed by KAPA Hyper Plus kit. DNA fragments captured by probes were pooled and sequenced using the Illumina HiSeq X TEN or NovaSeq 6000 platform, 1000× median coverage for tumour gDNA and 400× median coverage for blood gDNA, thereby eliminating germline variant interference and ensuring somatic mutations. Clinicopathological characteristics, including age, menopause, tumour size (T), lymph node status (N), metastasis status (M), histological grade, tumour histologic type and ER, PR, HER2 status were all recoded. Categorical variables of TNM and stage were strictly categorised according to the 8th American Joint Committee on Cancer (AJCC) staging manual.^24^ This study was approved by the Independent Ethical Committee of FUSCC, and all the participants signed written informed consent.

### 2.2 Reagents and antibodies

Lapatinib (Selleck Chemicals, #S2111), alpelisib (BYL719, Selleck Chemicals, #S2814) and cobimetinib (GDC‐0973, Selleck Chemicals, #S8041) were dissolved in DMSO (Sigma, #D2650) for in vitro studies and in 0.5% CMC‐Na (Selleck Chemicals, #S6703) for in vivo studies. The primary antibodies used for western blotting included those against p‐HER2‐Tyr1221/1222 (Abcam, #ab131102, 1:500), HER2 (CST, #2242, 1:1000), pIGF‐1R (CST, #3021, 1:500), IGF‐1R (CST, #9750, 1:500), p‐AKT‐Ser473 (CST, #4060, 1:1000), p‐AKT‐Thr308 (CST, #13038, 1:1000), AKT (CST, #4691, 1:1000), p‐ERK‐Tyr202/204 (CST, #4370, 1:1000), ERK (CST, #4695, 1:1000), p‐MEK‐Ser217/221 (CST, #9154, 1:1000), MEK (CST, #9126, 1:1000), p‐S6‐Ser240/244 (CST, #5364, 1:1000), S6 (CST, #2217, 1:1000), HA‐tag (CST, #3724, 1:1000), p110α (CST, #4249, 1:1000) and vinculin (CST, #13901, 1:2000), and HRP‐conjugated goat‐anti‐rabbit secondary antibody (Invitrogen, #31460, 1:5000). Exposure time of each band depends on the saturation degree of chemiluminescent, generally no more than 1 min.

### 2.3 Cell culture

Human breast cancer cell lines (SK‐BR‐3, AU565, BT474, MDA‐MB‐361, EFM192A, HCC1954, JIMT1, and ZR‐75‐30) were obtained from the American Type Culture Collection (ATCC) and were all cultured in high‐glucose DMEM (Gibco) supplemented with 10% foetal bovine serum (Gibco), and 100 μg/ml penicillin/streptomycin (Invitrogen). MCF‐10A and MCF‐10CA1a cells were obtained from the Shanghai Cell Bank Type Culture Collection Committee and cultured in DMEM/F12 (Gibco) with 10% horse serum (Gibco), 10 μg/ml insulin (Sigma), 100 μg/ml penicillin/streptomycin (Invitrogen), 0.5 μg/ml hydrocortisone (Sigma), 20 ng/ml EGF (Invitrogen) and 1 ng/ml cholera toxin (Sigma). All cell lines were mycoplasma negative and passed short‐tandem repeat (STR) test verification (Supplementary Data).

**HIGHLIGHTS**

1. Large‐scale Chinese breast cancer clinical sequencing.
2. *PIK3CA* mutations improved HER2‐positive patients’ disease‐free survival, but confer anti‐HER2 resistance in locally advanced/advanced cohorts.
3. Functional *PIK3CA* genomic alterations were annotated accurately via pooled library screening.
4. PI3Kα inhibitor was not necessary for treatment‐naïve HER2+ patients, whereas double‐blockade of mutated‐PI3Kα and enriched‐HER2 offered therapeutic potential for advanced patients.

### 2.4 Animal models

All the animal experiments complied with the performing protocols approved by the Research Ethical Committee of FUSCC. Six‐week‐old female NOD.CB17‐Prkdc^scid^/NcrCrl mice (NOD/SCID; Charles River) were injected with 5 × 10^6^ MCF‐10CA1a‐HER2 overexpressing cells harbouring *PIK3CA^WT^* and *PIK3CA^H1047R^*. After subcutaneous inoculation, mice were monitored daily and weighed, and tumours were measured every 3 days (volume = width^2^ × length/2). Once tumours reached 100 mm^3^, mice with *PIK3CA^H1047R^* were randomly divided into the following four treatment groups (*n* = 6 to 8 per group): vehicle control, 100 mg/kg lapatinib, 25 mg/kg BYL719, or 100 mg/kg lapatinib and 25 mg/kg BYL719. All drugs were administered orally every day (Figure 6D). Mice were sacrificed when showing signs of distress or tumour haemorrhage (3 weeks of drug treatment), and tumours were harvested, weighed, and photographed. Representative portions were stained with haematoxylin and eosin (H&E), or used for immunohistochemistry (IHC) of p‐HER2‐Tyr1221/1222 (CST, #2243, 1:320), HER2 (CST, #4290, 1:800), IGF1R (CST, #14534, 1:400), p‐AKT‐Ser473 (CST, #4060, 1:100), p‐ERK‐Tyr202/204 (CST, #4370, 1:400), HA‐tag (CST, #3724, 1:1600) and Ki67 (CST, #12202, 1:400).

### 2.5 Patient‐derived organoids

All tissue collections and experiments were performed in compliance with protocols approved by the Research Ethical Committee of FUSCC. Culture conditions and reagents for human mammary epithelial organoids were based on those previously described by Hans Clevers.^25^ Breast cancer tissues were pulverised and enzymatically digested with collagenase (Type I, III: Worthington, #LS004197, #LS004183) and hyaluronidase (Sigma, #H3884) on an orbital shaker at 37°C for 16 h. The digested tissue suspension was sequentially centrifuged at 500 *g*, at 4°C, for 5 min. Then we aspirated the supernatant and removed red blood cells by lysis buffer (Absin, #Abs9101); then, the pellet was resuspended in breast cancer organoid medium (BCOM: Advanced DMEM/F‐12 reduced serum media [ThermoFisher, #12634010] supplemented with GlutaMAX [ThermoFisher, #35050061], HEPES [ThermoFisher, #15630080], Primocin [InvivoGen, #ant‐pm‐2], B‐27 [ThermoFisher, #17504044], N‐acetylcysteine [Sigma, #A9165], β‐Estradiol [Sigma, #E2785], Nicotinamid [Sigma, #N0636], Noggin [Peprotech, #120‐10C], Neuregulin [Peprotech, #100‐03], Y27632 [Selleck, #S1049], FGF10 [Peprotech, #100‐26], FGF7 [Peprotech, #100‐19], EGF [Peprotech, #AF‐100‐15], R‐spondin1 [Peprotech, #120‐38], SB‐202190 [Sigma, # S7067], A83‐01 [Tocris, #2939]) and growth factor‐reduced Matrigel Type II (Trevigen, #3533‐010‐02) on ice. Next, 200 μl of the mixture of Matrigel and BCOM at a ratio of 1:1.5 was added into the lower chamber of 12‐well suspension culture plates (Corning) and placed in 37°C incubator for approximately 10 min until the mixture solidified. Then, 350 μl of the mixture of the Matrigel and organoids was added into the upper chamber and 1 ml of BCOM was added to each well. When more than 50% of the organoid sphere diameter reached 100 μm, the drug intervention was started. Organoids were harvested and diluted to an appropriate concentration, and 50 μl organoid suspension was added to each well. After one week, BCOM containing lapatinib (0.4 μM) and BYL719 (2 μM) at a concentration of twice the intracellular IC50 was added to each well in duplicate for five days, and photos were taken every day to observe the changes in organoids during drug treatments.

### 2.6 Construction and screening of the *PIK3CA* library

*PIK3CA library construction*. Retroviral vectors contained various barcodes referred to the previous description and each *PIK3CA* mutant was labelled with a unique 30‐bp barcode.^26^ We transformed the retroviral pDEST‐HA‐Flag backbone of the *PIK3CA* library into the lentiGuide‐pCDH‐puro backbone using the ClonExpress II One Step Cloning Kit (Vazyme, #C112). We also added 23 new *PIK3CA* mutant sites based on the precise sequencing results using the KOD‐Plus‐Mutagenesis Kit (TOYOBO, #SMK‐101). The pooled *PIK3CA* library contained 119 *PIK3CA* mutations, 2 *PIK3CA*‐WT and 2 control plasmids of *PIK3CA*‐GFP, which were mixed in equal amounts to infect SK‐BR‐3 at a low multiplicity of infection (MOI = 0.3), making sure that each transduced cell would express only a single *PIK3CA* variant with high probability. The stably integrated cells were selected with puromycin for 5 days.

*PIK3CA library screening*. On the first day of phenotype screening, cells were divided into three groups, with each group consisting of four replicates. For cell proliferation screening, cells were harvested and gDNA was extracted at Day 1 and Day 14 by a QIAamp DNA Mini Kit (Qiagen). For drug response screenings, cells were subjected to treatments with lapatinib or BYL719 for 14 days, and gDNA was sampled on Day 14. The relative abundance of each variant at the specific time point was assessed using Illumina sequencing and each sample was amplified and prepared through the following two‐step PCR procedures.^27^ First, the template gDNA of first‐round PCR was 2 μg (6.6 pg gDNA per cell × 123 clones × 2000) to ensure at least 2000 × coverage over the *PIK3CA* library, and then we amplified the barcode region for 20 cycles using NEBNext High‐Fidelity 2X PCR Master Mix (NEB, #0541). Primers were designed according to the Nextera^®^ transposase sequences (Illumina):

F1: TCGTCGGCAGCGTCAGATGTGTATAAGAGACAGaaatggattggatcttccac
R1: GTCTCGTGGGCTCGGAGATGTGTATAAGAGACAGtgggaaaagcgcctccccta

Second‐round PCR of seven cycles was performed to ligate adaptors and unique indices to each sample, and primers were designed on the basis of the Nextera^®^ Index Kit (Illumina):

F2: AATGATACGGCGACCACCGAGATCTACAC (I5 index) TCGTCGGCAGCGTC
R2: CAAGCAGAAGACGGCATACGAGAT (I7 index) GTCTCGTGGGCTCGG

Second‐round PCR products were purified with AMPure XP beads (Beckman Agencourt) and sequenced using the Miseq platform (Illumina).

*Sequencing data analysis*. The read counts of *PIK3CA* mutations represented by each barcode were obtained through Geneious 7.0 (Biomatters Inc.) and normalised according to the formula = reads per barcode/total reads of all barcodes × 10^6^ + 1. To determine the relative changes in clonal abundance, we compared the read counts corresponding to each unique barcode at Day 14 to the faction at Day 1. To determine the drug response, we compared Day 14 treatment read counts to those of Day 14 proliferation in order to eliminate confounding factors of proliferation.

### 2.7 Generation of HER2 overexpression plasmids and cells

We cloned the 3768‐bp HER2 fragment from a previously synthesised GV219 vector in the laboratory^28^ and inserted it into the pDONOR223 vector backbone using the gateway BP Clonase Kit (Invitrogen, #11789100). Hereafter, the construct was displaced and attached to the pDEST vector backbone by the gateway LR Clonase Kit (Invitrogen, #11791100). After Sanger sequencing, the HER2 overexpression plasmid and the envelope VSV‐G and gag‐pol packaging plasmids were introduced into 293T cells to generate retrovirus particles. MCF‐10CA1a cells were infected with virus for 2 days, after which cells were selected with 10 μg/ml blasticidin (Gibco, #A113903) for 5 days.

### 2.8 Western blot

Cells were serum‐starved overnight or treated with different drugs for a certain period of time. In case of detection of phosphorylation of IGF‐1R, cells were pre‐treated with the respective drugs for 2 days and then received insulin stimulation (100 nM) (BasalMedia, #S450J7) for 30 min. Whole‐cell protein was obtained with Tissue Protein Extraction Reagent (Thermo Fisher Scientific, #78510), protease and phosphatase inhibitors (CST, #5872) for 30 min; lysates were then centrifuged at 12 000 g for 15 min at 4°C and protein was quantified by BCA protein assay (Solarbio, #PC0020). 25 μg protein samples and the 5 × loading buffer (Biosharp, #BL502A) were loaded, separated by electrophoresis, transferred to PVDF membranes (Invitrogen, #T2234) and incubated with the primary and secondary antibody prospectively, and the protein–antibody blots were detected using chemiluminescence substrate ECL (Thermo Fisher Scientific, #34577).

### 2.9 qPCR

RNA of *PIK3CA*‐WT and *PIK3CA*‐H1047R from SK‐BR‐3 cells was harvested by RNeasy Mini Kit (Qiagen) and reverse‐transcribed to cDNA according to the PrimeScript RT Reagent Kit (TaKaRa, #RR037A). Synthesised cDNA was performed on 7300 Real‐Time PCR System with SYBR qPCR Master Mix Kit (Vazyme, #Q311‐02). Samples were normalised to GAPDH and analysed by the 2^−△△Ct^ method. Primers were designed with PrimerBank and detailed sequences are shown in Table S9.

### 2.10 Cell viability studies and combination index analysis

For the cell viability studies, 4 × 10^3^ cells at the logarithmic stage of growth were plated in 96‐well plates and treated with respective concentrations of drugs for 5 days. Ten microlitres of CCK‐8 solution (Dojindo, #CK04) was added to each well and incubated for 2 h at 37°C, and the absorbance was determined at 450 nm. The nonfixed ratio was used with cellular viability measurements over two‐dimensional gradients of BYL719 and lapatinib or GDC‐0973, such that sequentially diluted concentrations of BYL719 (8, 4, 2, 1, 0.5 μM) were added with fixed concentrations of lapatinib (8, 4, 2, 1, 0.5 μM) or GDC‐0973 (6, 3, 1.5, 0.75, 0.375 μM) for three days. CompuSyn used the median effects method to calculate the combination index (CI), of which values greater or less than 1 indicate antagonism and synergism.

### 2.11 Statistical analysis

Associations between the clinical information and *PIK3CA* mutation status were analysed by Pearson's chi‐square test. Statistical differences in cell proliferation, IC50 values, RTK transcriptome expression, tumour volume and weight were determined by Student's t test and one‐way ANOVA . Survival analysis was analysed by the Kaplan–Meier test and the log‐rank test. The criteria used to determine objective tumour response were as follows: complete response (CR): disappearance of all target lesions and lymph nodes < 10 mm; partial response (PR): ≥30% decrease in the sum of the diameters of the target lesions; progressive disease (PD): < 20% increase in diameters of all target lesions or the appearance of one or more new lesions; stable disease (SD) with neither sufficient shrinkage to PR nor increase to PD; overall response (OR) defined as the sum of the rates of PR and CR. All *p* values were two‐sided, and *p *< .05 was considered significant. All analyses were performed using R package version 4.0.0 and GraphPad Prism 7.

## 3 RESULTS

### 3.1 Patient demographics and clinical–pathological characteristics

We are conducting a prospective clinical sequencing program for patients diagnosed as breast cancer at FUSCC.^21^ In this study, we included 1923 breast cancer patients consisting of 1140 primary breast cancer patients receiving surgical treatment (cohort 1, early), 604 locally advanced patients receiving neoadjuvant therapy (cohort 2, neoadjuvant), and 179 advanced breast cancer patients (cohort 3, advanced, 105 with initial stage IV and 74 with tumour recurrence or metastasis) who received systemic treatments. The patients’ clinical–pathological information was listed in Table S2. In this FUSCC‐BC prospective cohort, Luminal A (*n* = 375) and Luminal B (HER2‐, *n* = 559) subtypes account for 40%, which are lower than those in the MSKCC cohort; meanwhile, the FUSCC‐BC cohort includes more patients with Luminal B (HER2+, 13.52%, *n* = 260), HER2+ (18.46%, *n* = 355) and TNBC (19.03%, *n* = 366), respectively (Figure S1A).

### 3.2 Genomic alterations in the RTK‐PI3K‐MAPK signalling in the FUSCC‐BC cohort

**FIGURE 1:**
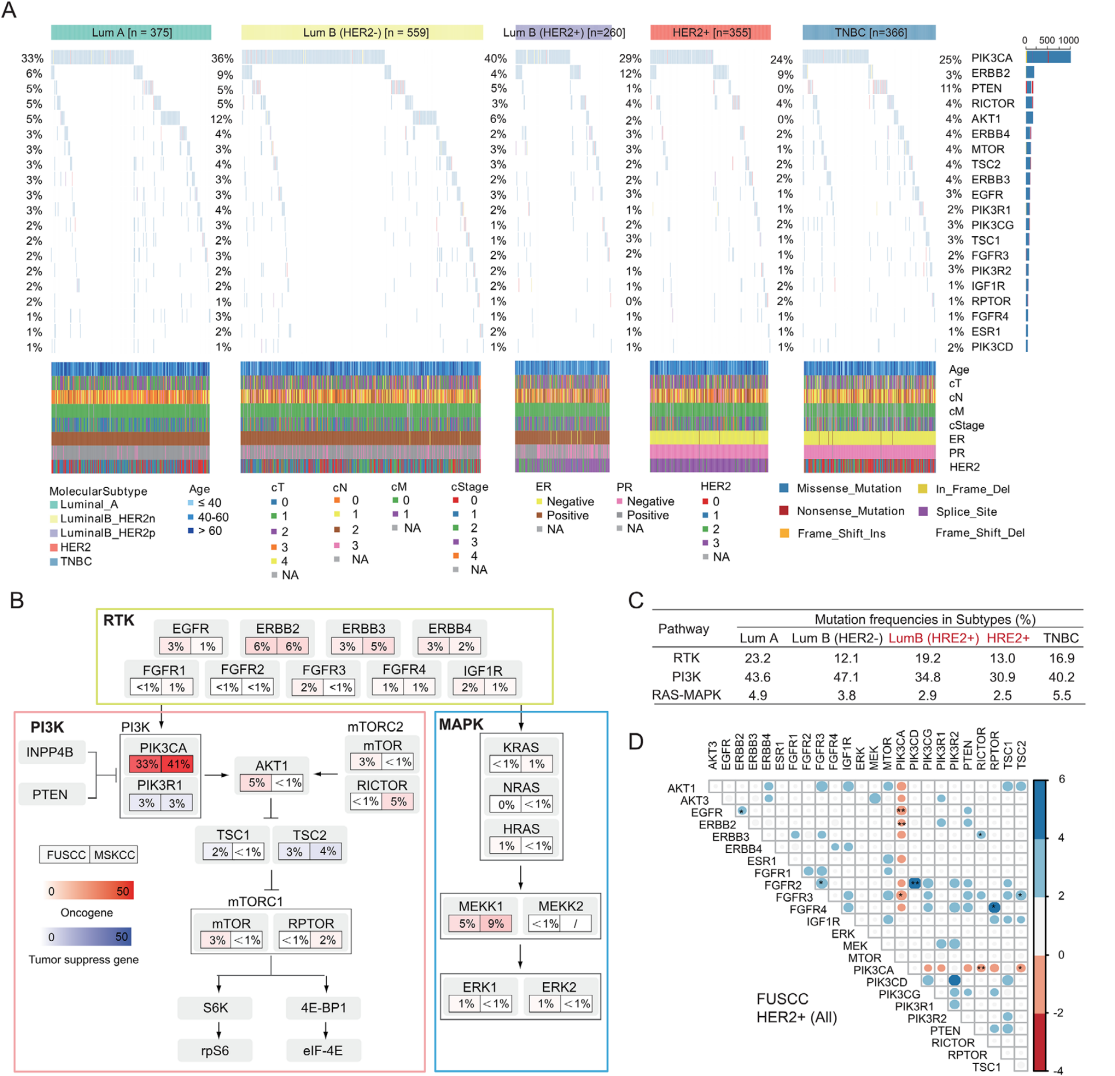
Genomic characteristics of the RTK‐PI3K‐MAPK pathway in the FUSCC cohort. (A) The genomic landscape of alterations among RTK‐PI3K‐MAPK pathway in FUSCC breast cancer sequencing cohort classified by molecular subtypes. (B) A comparison of somatic alteration frequencies for the RTK‐PI3K‐MAPK‐related genes between the FUSCC and MSKCC cohorts. (C) Overall alteration frequency of the RTK, PI3K and MAPK pathways per subtype. (D) Mutual co‐occurrence (blue) and mutual exclusivity (red) of gene mutations among the RTK‐PI3K‐MAPK pathway in the FUSCC HR±HER2+ breast cancer cohort. Scale bar: value of log10 ratio of odds ratio (OR); value scaled with colour intensity. * *p* < .05, ** *p* < .01, *** *p* < .001

The most frequently somatic altered gene in RTK‐related pathways was *PIK3CA* (33%), and this frequency was lower than that in the MSKCC cohort (41%). The *PI3KCA* mutations were prevalent in luminal B (HER2–) (40%) and luminal A subtypes (36%) in Chinese patients, while the proportions of these two subtypes in the FUSCC‐BC cohort were lower than those in the MSKCC cohort (Figure 1A, B). We also evaluated the frequencies of oncogenic alterations in RTK, PI3K and MAPK signalling pathways (Figure 1C, Table S3). We found a higher mutation frequency in the HER2– subgroups (luminal A, 43.6%; luminal B [HER2–], 47.1%; and TNBC, 40.2%) and a lower mutation frequency in the HER2+ subgroups (luminal B [HER2+], 34.8%; and HER2+, 30.9%), demonstrating the disparity PI3K mutational features among different subtypes.

Notably, missense mutations in *PIK3CA* were illustrated in a mutually exclusive pattern with other somatic alterations in its up‐ and down‐stream molecules, including *EGFR, ERBB2, ERBB3, FGFR2, FGFR3, FGFR4, AKT1, AKT3* and *ESR1* in HER2+ breast cancer (Figure 1D). The similar trends were observed in other subtypes, regardless of hormone receptor status in the FUSCC or MSKCC cohorts (Figure S2A–G). Compared with those in the MSKCC cohort, the frequencies of well‐known *PIK3CA* driven mutations in our cohort were higher for p.H1047R (44% vs. 38%) and lower for p.E545K (7% vs. 22%), p.E542K (2% vs. 14%), and p.N345K (4% vs. 8%), which were consistent with the findings of Liao et al in anther Chinese breast cancer cohort^30^ (Figure S3A).

Given that mutated‐PI3Kα can continuously activate downstream signalling and be mutually exclusive with other kinases, we will be interesting to evaluate the clinical influence of *PIK3CA* mutations in HER2+ subgroup in which the HER2 signalling is hyperactive. The frequency of *PIK3CA* mutations were the highest in cohort 1 (early) regardless of the HR status (HER2+_all, 33.12%; HR+HER2+, 30.77%, HR‐HER2+, 36.30%), followed by cohort 2 (neoadjuvant) of an approximate 25% and advanced cohort 3 (HER2+_all, 25.00%; HR+HER2+, 21.43%, HR‐HER2+, 26.92%) (Figure S3B).

### 3.3 *PIK3CA* mutations correlate to improved disease‐free survival for HER2+ patients, but confer anti‐HER2 resistance in advanced cohorts

Although the numerous studies have defined *PIK3CA* mutations as oncogenic alteration, the prognostic value of *PIK3CA* mutations in HER2+ breast cancer patients is still ambivalent. As our patients were mainly recruited from April 2018 to September 2019, and the insufficient follow‐up might limit the significance of a survival analysis, we performed survival analysis using the dataset from the MSKCC cohort. Although there was no OS difference between patients with *PIK3CA* mutations and *PI3KCA*‐WT (HER2+, *p *= .9; HR+HER2+, *p *= .8; HR‐HER2+, *p *= .6, Figure S4A), we unexpectedly found that *PIK3CA* mutations indicated a significantly lower risk for disease relapse than *PI3KCA*‐WT in disease‐free survival (DFS) analysis (HER2+, *p *= .005; HR+HER2+, *p *= .01; HR‐HER2+, *p *= .1, Figure S4B).

**FIGURE 2:**
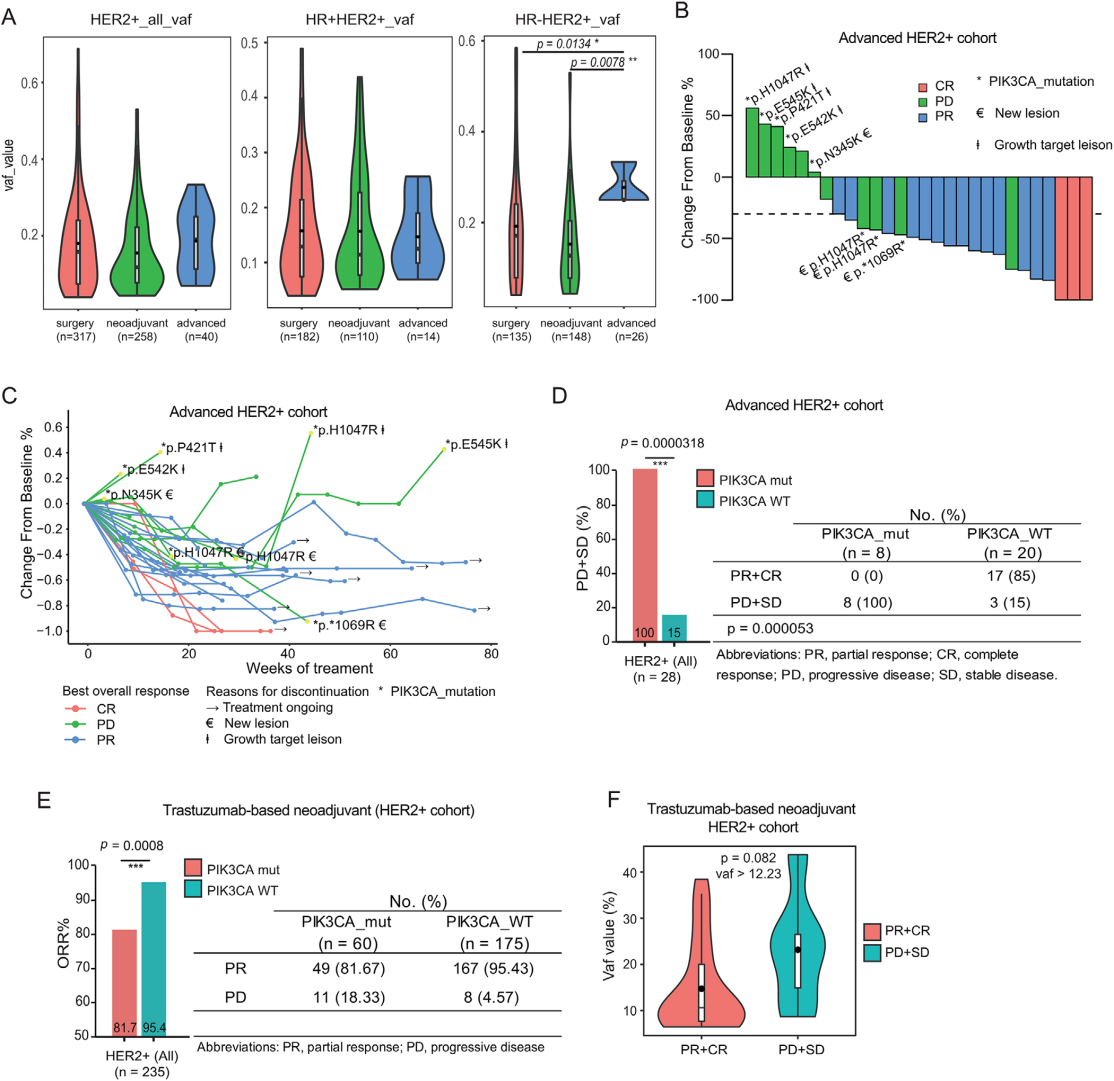
*PIK3CA* mutations enriched in advanced HER2+ breast tumours and associated with worse efficacy of anti‐HER2 therapy for locally advanced or advanced HER2‐positive patients. (A) The variant allele frequency (VAF) of *PIK3CA* mutations increases among advanced HER2+ subgroup (left, HER2+ all cases; middle, HR+HER2+ cases; right, HR‐HER2+ cases). The efficacy of trastuzumab‐based therapy in advanced HER2+ patients is shown in the waterfall plot (B) and the spider plot (C). The *y*‐axis represents the percentage of maximum tumour reduction compared to the baseline data. (D) Statistical analysis of the progressive response (PD+SD) for patients carrying *PIK3CA*‐WT or *PIK3CA* mutations in the advanced HER2+ cohort using a fisher's exact test. (E) Objective response rate according to *PIK3CA* mutation status in locally advanced HER2+ patients receiving neoadjuvant therapy, using a χ^2^ test of association. (F) Comparison of VAF distribution between the PR+CR and PD+SD groups in HR‐HER2+ breast cancer patients harbouring *PIK3CA* mutation (VAF threshold > 12.23%). OR, overall response; CR, complete response; PR, partial response; PD, progressive disease

In the FUSCC cohort, we found *PIK3CA* mutations were like to enriched in the metastatic HR‐HER2+ tumours which were exposed with long‐term trastuzumab‐based treatment via the analysis of variant allele fraction (VAF) (*p *= .0078, Figure 2A). Subsequently, we further explored the roles of *PIK3CA* mutations in the advanced HER2+ cohort who received trastuzumab‐based clinical treatment. We analysed 28 cases of primary stage IV HER2+ breast cancer patients from cohort 2. The treatment response was assessed in accordance with the RECIST 1.1 criteria.^30^ The waterfall and spider plots showed that 3 patients achieved CR, 14 patients had PR, but none of these 17 patients harboured the *PIK3CA* mutation (Figure 2B, C). The other 11 patients had progressive disease (PD), and eight of them harboured *PIK3CA* mutations, which indicated that the anti‐HER2 treatment effectiveness was limited for advanced HER2+ breast cancer patients with *PIK3CA* mutations than for those with *PIK3CA*‐WT (100% vs. 15%, *p = *.000053, Figure 2D).

We further analysed the effects of *PIK3CA* mutations in the locally advanced HER2+ patients (235 cases) from cohort 3 who had received trastuzumab‐based neoadjuvant chemotherapy. The primary endpoint was the overall response rate (ORR). *PIK3CA* mutations were associated with a significantly lower ORR than *PI3KCA*‐WT (81.7% vs. 95.4%, *p *= .0008, Figure 2E). In the HR‐HER2+ subgroup, the ORR decreased from 96.9% of *PIK3CA*‐WT patients to 78.8% of patients harbouring *PIK3CA* mutations (*p *= .0007), and the same trend also existed in the HR+HER2+ subgroup (85.2% vs. 93.4%, *p *= .193, Figure S5A, Table S4A). We further defined a VAF threshold using a receiver operating characteristic (ROC) curve and found that the cut‐off value was 12.23% (Figure S5B, C), at which point the *PIK3CA* mutation was a predictor of reduced‐effectiveness in response to neoadjuvant anti‐HER2 treatment in HR‐HER2+ subgroup (*p *= .082, Figure 2F, Table S4B). The above clinical analyses demonstrated a pleiotropic effect of *PIK3CA* mutations which were stage dependent, *PIK3CA* mutations were associated with improved DFS in all HER2+ breast cancer patients, but served as an indicator of poor response to continuous trastuzumab‐based treatments in locally advanced or advanced HER2+ cohorts.

### 3.4 Pooled mutation‐barcoding screens reveal *PIK3CA* functional mutations determine proliferation ability and drug responses in HER2+ breast cancer

**FIGURE 3:**
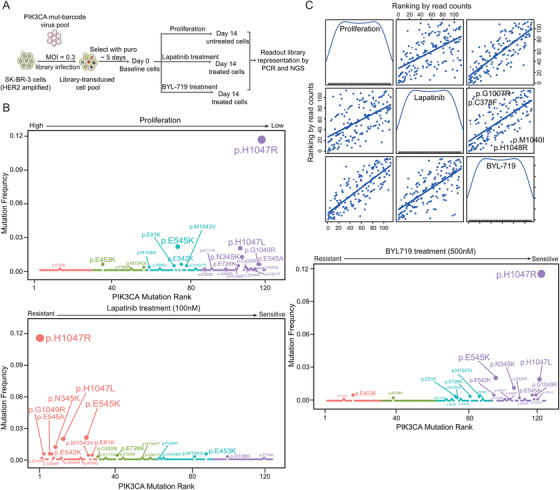
Pooled mutations‐barcoding screens for functional *PIK3CA* mutations relating to oncogenesis and drug responses. (A) Schematic representation of the *PIK3CA* mutations‐barcoding screens. (B) *PIK3CA* mutations are ranked by the normalised read count ratio corresponding to proliferation (Left upper panel) and responses to lapatinib (Left down panel) and BYL719 (Right panel). (C) Two‐dimensional scatterplot matrix of the ranking of *PIK3CA* mutation sites between proliferation, lapatinib and BYL719 treatment groups. In each box, the diagonal lines above (the upper left corner) indicate a suppression of proliferation and sensitivity to both lapatinib and BYL719, which is opposite in the lower right corner

We further launched biologic exploration to explain the contradictory roles of *PIK3CA* mutations. Pooled mutation‐barcoding library, containing 119 *PIK3CA* mutations and controls (Table S5), were conducted to achieve precise annotation of impactful *PIK3CA* mutations that determine the proliferation ability of HER2+ mammary cancer cells and drug responses under different treatments (Figure 3A). To select a suitable cell line for library transduction, we performed the whole exome sequencing (WES) on eight HER2+ cell lines with the following criteria: (1) *PIK3CA* and *ERBB family* have no functional missense mutations, avoiding promoting oncogenic signalling and interfering the direct and bypass pathways^31^, ^32^ and (2) the RTK/PI3K pathway is activated by amplified‐*ERBB2* and can be blocked by the TKI lapatinib and neratinib (Figure S6A). Finally, we chose SK‐BR‐3 as the candidate cell line for exogenously introduced *PIK3CA* ReMB library. NGS was performed to analyse dynamic changes in specific barcodes in different samples. The barcode read counts and the correlation index showed high concordance within the replicates and apparent diversity between different groups (Figure S6B, C). The cumulative frequency curve indicated that the mutant distributions were strongly distinct between baseline Day 0 and endpoint Day 14 (Figure S6D).

We ranked the average normalised barcode read counts. A higher ranking indicated that *PIK3CA* mutations enriched in proliferation and drug treatment; in contrast, a lower ranking represented the clonal disadvantage of cells carrying *PIK3CA* mutations. We found that most *PIK3CA* hotspot mutations (H1047R/L, E542K, E545K and N345K) were shown to suppress the proliferation of HER2+ cancer cells (Figure 3B left upper panel and Table S6), while these mutations exhibited a higher tolerance to lapatinib treatment (Figure 3B left down panel and Table S7). Importantly, compared with *PIK3CA* ^WT^, the *PIK3CA*functional mutations were more sensitive to the PI3Kα‐specific inhibitor BYL719 in HER2+ cells (Figure 3B right panel and Table S8). Additionally, we found some mutation sites were resistant to lapatinib and BYL719 simultaneously, like p.H1048L, p.M1040I and vice versa, sites like p.G1007R and p.C378F were sensitive to both lapatinib and BYL719 (Figure 3C).

### 3.5 *PIK3CA* functional mutations inhibit HER2+ tumour proliferation via suppressing HERs and RTKs through negative feedback

**FIGURE 4:**
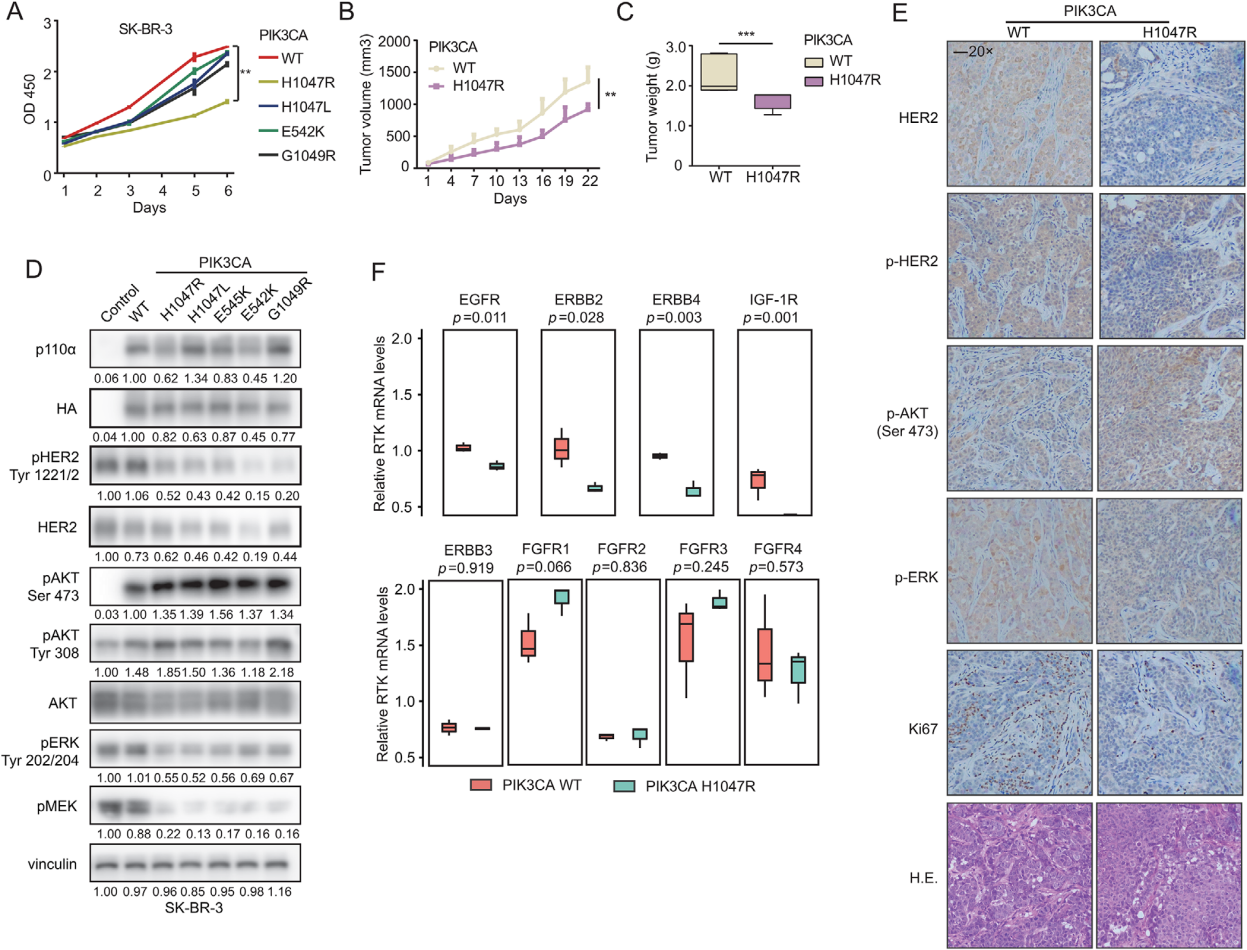
The *PIK3CA* functional mutations mediate the negative feedback inhibition of RTKs which contributes to suppression of HER2+ cells proliferation. (A) Proliferation viability of SK‐BR‐3 cells expressing *PIK3CA* wild‐type and mutants using a CCK‐8 assay. Xenograft tumour volumes (B) and tumour weights (C) of mice bearing *PIK3CA* ^WT^ and *PIK3CA* ^H1047R^ cells. Error bars, mean ± SEM (*n* ≥ 5 per treatment group). (D) Immunoblot detecting activation of the key molecules among RTK‐PI3K‐MAPK pathway in HER2+ cells harbouring pCDH‐NC*, PIK3CA‐*WT and mutants. Control referred to SK‐BR‐3 with empty plasmid pCDH (pCDH‐NC). (E) Tumour tissues of mice bearing *PIK3CA* ^WT^ and *PIK3CA* ^H1047R^ cells were harvested and subjected to IHC analysis for HER2, phospho‐HER2, phospho‐AKT (Ser473), phospho‐ERK, Ki67 and H&E staining. (F) Expression levels of RTK transcripts were examined by quantitative PCR (qPCR) in SK‐BR‐3 cells carrying *PIK3CA* mutant and control. The results are shown as the mean ± SD of three independent experiments

To validate the functional impact of *PIK3CA* mutations, several top‐ranking *PIK3CA* mutations were stably introduced in SK‐BR‐3 cells, while the wild‐type sequence served as a control. Consistent with the results of the pooled screen, all these *PIK3CA* functional mutations suppressed SK‐BR‐3 cell proliferation, and the growth curve of *PIK3CA* ^H1047R^ was significantly lower than *PIK3CA* ^WT^ (*p *< .05) (Figure 4A). As expected, the tumour volume of *PIK3CA* ^H1047R^ was significantly smaller than that of *PIK3CA* ^WT^ (*p *= .0012), as was the tumour weight (*p *= .0079) in the in vivo xenograft model (Figures 4B, C and S9A).

Compared to *PIK3CA* ^WT^, the expression levels of phosphorylated AKT at both the Ser473 and Tyr308 residues were markedly upregulated in SK‐BR‐3 cells overexpressing the *PIK3CA* mutations (H1047R, H1047L, E545K, E542K and G1049R). These mutations induced the suppression of HER2 expression and phosphorylation through a negative feedback loop. ERK phosphorylation at Tyr204 and MEK phosphorylation at Ser221 were also markedly decreased in cells carrying *PIK3CA* mutations, which might also be mediated by the suppression of upstream HER2 signalling (Figure 4D). This negative feedback loop was also observed in the xenograft mouse model through IHC staining examination (Figure 4E).

To clarify that negative feedback occurs at the level of protein or RNA transcription, we compared the transcription levels of several RTKs in *PIK3CA* ^WT^ and *PIK3CA* ^H1047R^ cells by quantitative PCR. We noticed that the mRNA level of *EGFR* (*p *= .011), *ERBB2* (*p *= .028), *ERBB4* (*p *= .003) and *IGF‐1R* (*p *= .001) were significantly down‐regulated in *PIK3CA* mutant cells (Figure 4F), which is consistent with TCGA dataset with lower transcriptome expression level of *ERBB2* and *IGF‐1R* in HER2+ tumours harbouring *PIK3CA* H1047R mutations.

### 3.6 *PIK3CA* functional mutations mediate resistance to tyrosine kinase inhibitor (TKI), but sensitivity to PI3Kα‐specific inhibitor

**FIGURE 5:**
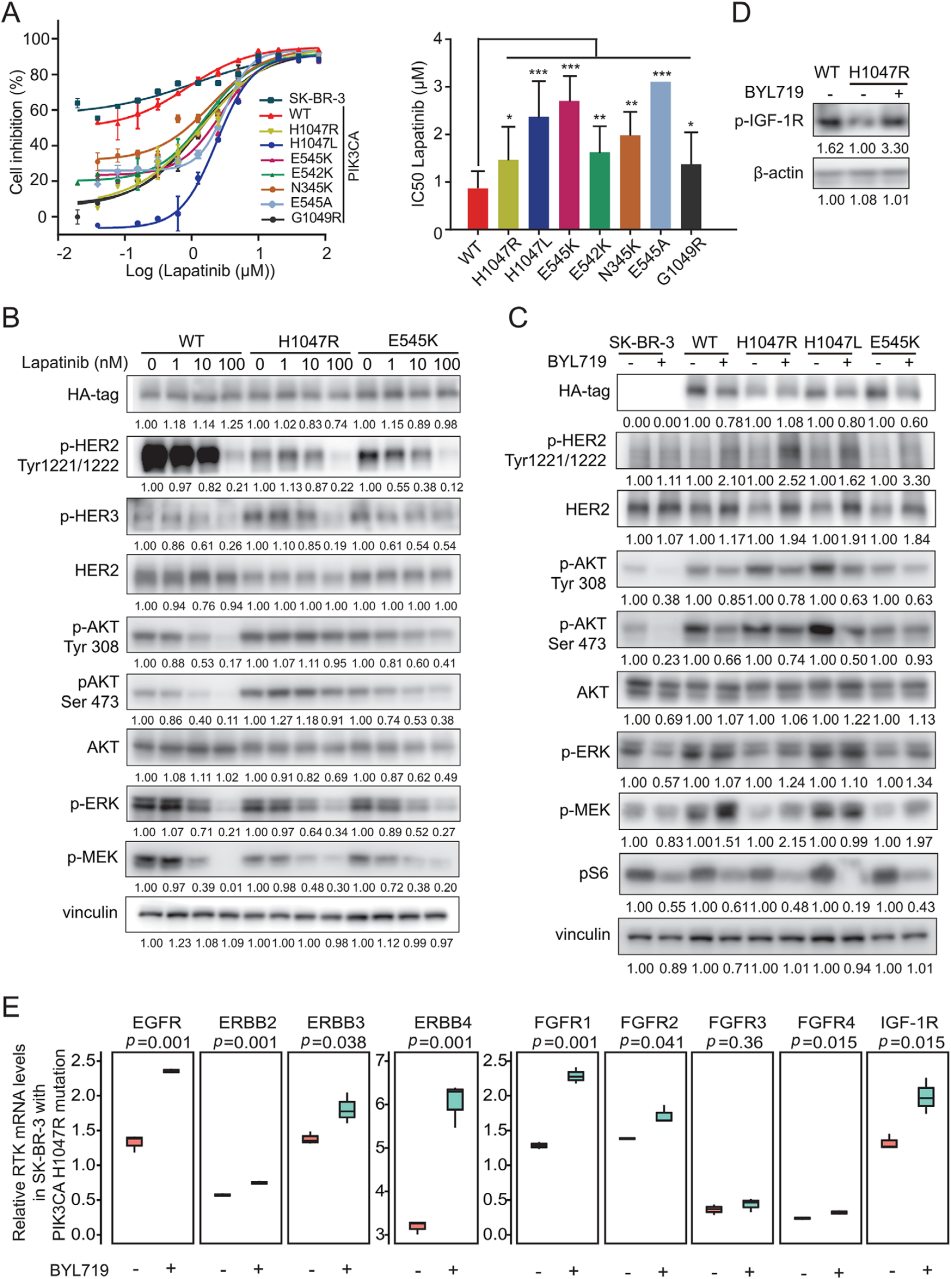
*PIK3CA* functional mutations induce drug resistance to TKI but sensitivity to PI3Kα‐specific inhibitor. (A) Responses of SK‐BR‐3 cells expressing *PIK3CA* wild‐type and mutants to different doses of lapatinib treatment by CCK‐8 assay. Half‐maximal inhibitory concentration (IC_50_) values were calculated after drug treatment for 5 days. Quantitative results of IC_50_ values are shown (right). (B) Activation status of RTK‐PI3K‐MAPK pathway in SK‐BR‐3 cells stably expressing *PIK3CA* ^WT^, *PIK3CA* ^H1047R^, *PIK3CA* ^E545K^, which were treated with the indicated concentration of lapatinib (1, 10, 100 nM) for 3 h. (C) Activation status of RTK‐PI3K‐MAPK pathway in SK‐BR‐3 cells stably expressing *PIK3CA* ^WT^, *PIK3CA* ^H1047R^, *PIK3CA* ^H1047L^ and *PIK3CA* ^E545K^, which were treated with 500 nM BYL719 for 2 days. (D) Immunoblot of pIGF‐1R in SK‐BR‐3 cells stably expressing *PIK3CA* ^WT^ and *PIK3CA* ^H1047R^ were treated with 500 nM BYL719 for 2 days followed by insulin stimulation (100 nM) for 30 min. (E) Transcriptional expressions of RTKs in HER2+ cells expressing *PIK3CA* ^WT^ or *PIK3CA* ^H1047R^ which were treated with BYL719 (500 nM, *n* = 3). Data are presented as the mean ± SD. *p < .05, **p < .01, ***p < .001

According to the readout of the *PIK3CA* mutation screen, we speculated that HER2+ mammary cancer cells harbouring *PIK3CA* mutations might exhibit resistance to lapatinib treatment. As expected, SK‐BR‐3 cells stably expressing *PIK3CA* functional mutations showed robust resistance to lapatinib compared to cells expressing *PIK3CA* ^WT^ (*p *< .05) (Figure 5A). To further explore the effect of mutated‐PI3Kα in response to lapatinib, SK‐BR‐3 cells harbouring *PIK3CA* ^WT^, *PIK3CA* ^H1047R^ and *PIK3CA* ^E545K^ were starved overnight and treated with increasing doses of lapatinib for 3 h. Western blots indicated that lapatinib could reduce HER2 phosphorylation in *PIK3CA* ^WT^ cells, consequently suppressing the phosphorylation levels of AKT and ERK, while mutated‐PI3Kα could constitutively activate downstream AKT signalling which bypassed the lapatinib‐induced blockade of the RTK‐PI3K pathway (Figure 5B).

Pooled screen also showed that cells carrying *PIK3CA* functional mutations were sensitive to treatment with PI3Kα inhibitor BYL719 (Figure S7). As illustrated in Figure 5C, the phosphorylation of AKT at the Tyr308 and Ser473 residues and subsequent phosphorylation of the S6 ribosomal protein were reduced by BYL719 treatment. Notably, HER2 expression and activation were upregulated by inhibition of PI3Kα, which could account for the blockage of negative feedback loop between *PIK3CA* functional mutations and HER2 signalling. As the *PIK3CA* ^H1047R^ mutation reduced the expression and activation of IGF‐1R, the blockage of the *PIK3CA* mutations by BYL719 could restore the activation of IGF‐1R signalling (Figure 5D). BYL719 upregulated multiple RTKs expressions at the transcriptional level (Figure 5E), suggesting that inhibition of hyperactive PI3K kinase played a major role in relieving the negative feedback loop (*EGFR*, *p *= .001; *ERBB2*, *p *= .001; *ERBB3*, *p *= .038; *ERBB4*, *p *= .001; *FGFR1*, *p *= .001; *FGFR2*, *p *= .041; *FGFR4 p *= .015; *IGF‐1R*, *p *= .015).

### 3.7 Blockage of mutated‐PI3Kα synergises with anti‐HER2 therapy to overcome drug resistance

Amplifed‐*ERBB2* in breast cancer activates downstream pathways including PI3K and MAPK signalling, and the mutated‐PI3Kα‐induced negative feedback may determine the success of anti‐HER2 therapy. In primary HER2+ breast cancer, continuously activating PI3K mutations play an inhibitory role to inhibit the expression and activation of HER2 and other RTKs; meanwhile, in advanced HER2+ cases, long‐term anti‐HER2 treatment resulted in the enrichment of PI3K mutant clones which eventually led to the failure of anti‐ HER2 therapy.

**FIGURE 6:**
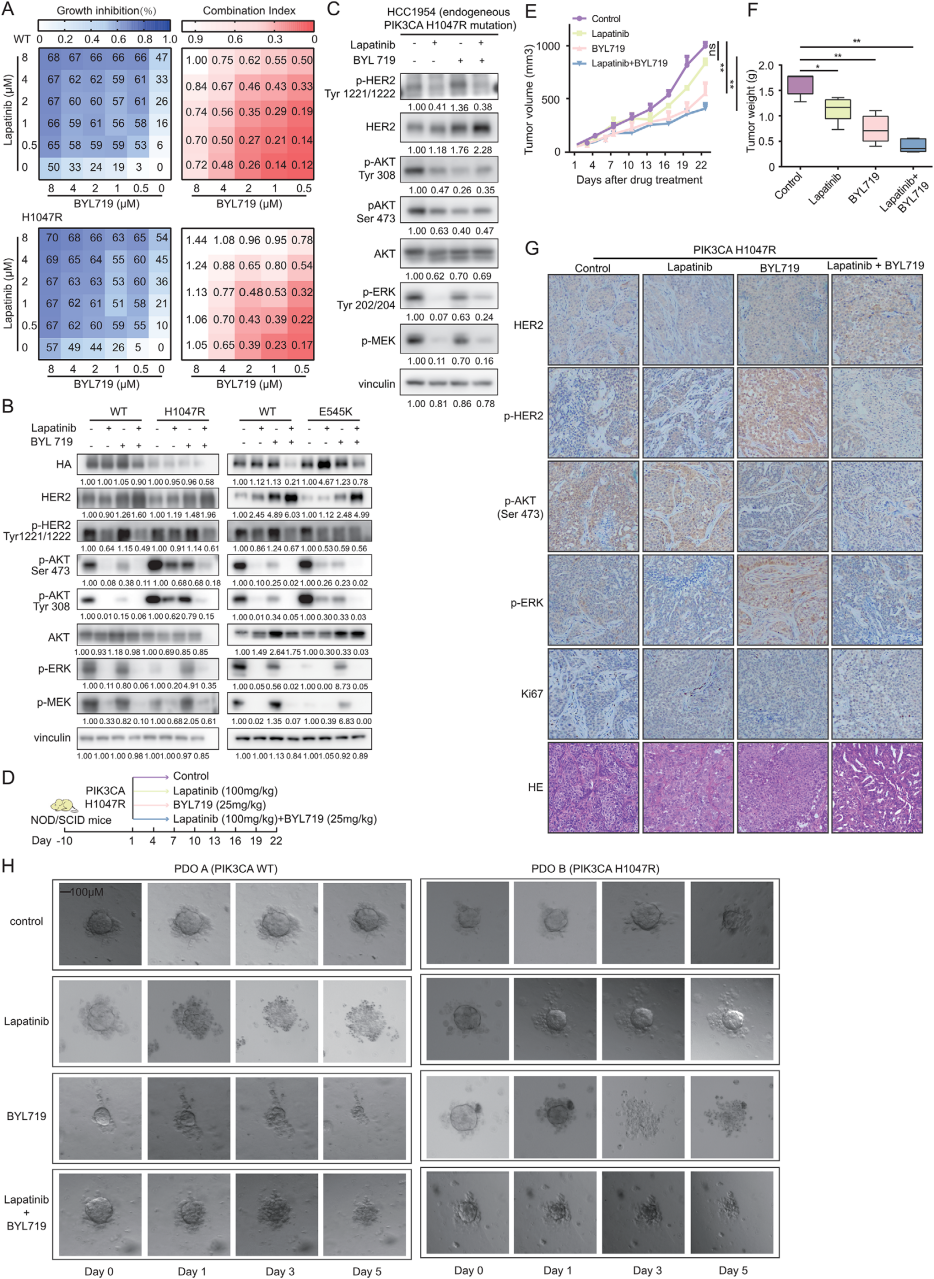
BYL719 synergises with lapatinib to suppress the proliferation of HER2+ cells harbouring *PIK3CA* mutation. (A) SK‐BR‐3 cells expressing *PIK3CA* ^WT^ and *PIK3CA* ^H1047R^ were treated with lapatinib, BYL719 or both as indicated. The percentage inhibition (left) and CI (right) at each concentration of the drugs are presented. Each score represents data from three independent experiments. (B) Activation status of the RTK‐PI3K‐MAPK pathway in *PIK3CA* ^WT^, *PIK3CA* ^H1047R^, *PIK3CA* ^E545K^ cells treated with lapatinib (500 nM), BYL719 (500 nM) and both. (C) Immunoblot analysis of key molecules among the RTK‐PI3K‐MAPK pathway in HER2+ breast cancer cell line HCC1954 with an endogenous *PIK3CA* ^H1047R^ mutation treated with lapatinib (500 nM), BYL719 (500 nM) and both. (D) Drug administration model for NOD/SCID mice bearing MCF‐10CA1a cells over‐expressing HER2 and *PIK3CA* ^H1047R^ mutant. Mice were randomly divided into the following four treatment groups (*n* = 6 to 8 per group): vehicle control, 100 mg/kg lapatinib, 25 mg/kg BYL719, or combination of 100 mg/kg lapatinib and 25 mg/kg BYL719. Tumour volumes (E) and tumour weights (F) of mice bearing *PIK3CA* ^H1047R^ cells treated with vehicle, lapatinib (100 mg/kg body weight), BYL719 (25 mg/kg body weight), or both drugs in combination for the indicated times. Error bars, mean ± SEM (*n* ≥ 5 per treatment group). (G) Tumour tissues of mice bearing *PIK3CA* ^WT^ and *PIK3CA* ^H1047R^ cells treated with vehicle, lapatinib (100 mg/kg body weight), BYL719 (25 mg/kg body weight), or both drugs in combination for 22 days was evaluated by IHC for HER2, phospho‐HER2, phospho‐AKT (Ser473), phospho‐ERK, Ki67 and H&E staining. (H) Clinical validation from patient‐derived organoids with wild‐type *PIK3CA* (PDO A) and mutated *PIK3CA* ^H1047R^ (PDO B) in the presence of the HER2 inhibitor lapatinib (0.4 μM) and the PI3K inhibitor BYL719 (1 μM) treated for 5 days. The same organoid's image were captured at Day 0, 1, 3, 5

Given the successful usage of PI3Kα inhibitor in luminal (HR+) breast cancer, we next tested whether a dual blockade strategy could effectively inhibit mutated‐PI3Kα and enriched‐HER2 simultaneously. As illustrated in the combination studies, combined treatment of 0.5 μM lapatinib and 0.5 μM BYL719 lead striking synergy in HER2+ cells carrying *PIK3CA* ^WT^ (CI = 0.12) and *PIK3CA* ^H1047R^ (CI = 0.17) (Figure 6A). Lapatinib counteracts BYL719‐induced increments of p‐HER2, p‐AKT, p‐ERK and p‐MEK effectively, and synergises with BYL719 in inhibiting proliferation of HER2+ cell exhibiting either exogenous or endogenous *PIK3CA* functional mutant (Figure 6B, C).

The combination of lapatinib and BYL719 also showed significant synergetic efficacy compared with single‐agent treatment in vivo. The treatment with lapatinib alone did not inhibit the growth of *PIK3CA* ^H1047R^ tumour transplants as expected (*p *= .3203), while a single dose of BYL719 could suppress *PIK3CA* ^H1047R^ tumour growth (*p *= .0043); in addition, significant tumour regressions were observed in the group treated with lapatinib and BYL719 (*p *= .0022) (Figures 6E and S9B). The tumour weight also showed similar trends (Figure 6F). Moreover, the immunohistochemistry staining of the *PIK3CA*‐mutant model showed the concomitant increments of phosphorylation of HER2, AKT, ERK after BYL719 treatment which could be reversed by lapatinib treatment (Figure 6G).

The PI3K inhibitor abolishes AKT activation, resulting in compensatory activation of the HER2/MAPK pathway, which may mediate the failure of BYL719. Therefore, we chose the MEK inhibitor GDC‐0973 as an alternative approach. This combination also showed the synergistic antitumour efficacy (Figure S8).

Patient‐derived organoid (PDO) is a robust model for preclinical and pharmaceutical research. We isolated two organoids from one patient with wild‐type *PIK3CA* (PDO A) and another patient with the *PIK3CA* ^H1047R^ mutation (PDO B), and validated their genomic status using targeted sequencing. Figure 6H illustrated the different viabilities of breast cancer organoids treated with the HER2 inhibitor lapatinib (0.4 μM), the PI3K inhibitor BYL719 (1 μM) or the combination of two drugs at Day 1, Day 3 and Day 5. BYL719 treatment abolished *PIK3CA* ^H1047R^‐mediated anti‐HER2 resistance, indicating that the combination of lapatinib and BYL719 provides a promising strategy to overcome the dual genomic carcinogen of mutated‐PI3Kα and enriched‐HER2 in breast cancer.

## 4 DISCUSSION

Taken advantage of the large‐scale clinical sequencing in this study, we redefined the adaptive roles of mutated‐PI3Kα in HER2+ breast cancer. *PIK3CA* mutations protect against the development of malignancy in early breast cancer, inhibiting the proliferation of HER2+ cells by the negative feedback of RTK expression and activation; however, *PIK3CA* mutations mediated a lower ORR in the trastuzumab‐based neoadjuvant or advanced breast cancers, and enriched in metastatic HER2+ tumours. The VAF value >12.23% could be used as a threshold to predict poor treatment efficacy in anti‐HER2 therapy. Among HER2‐positive (HER2+) breast cancers, patients with PI3K mutations account for a considerable proportion; thus, the accurate annotations of PI3K mutation in the disease progression will be critical for genomics‐guided diagnosis and treatment.

**FIGURE 7:**
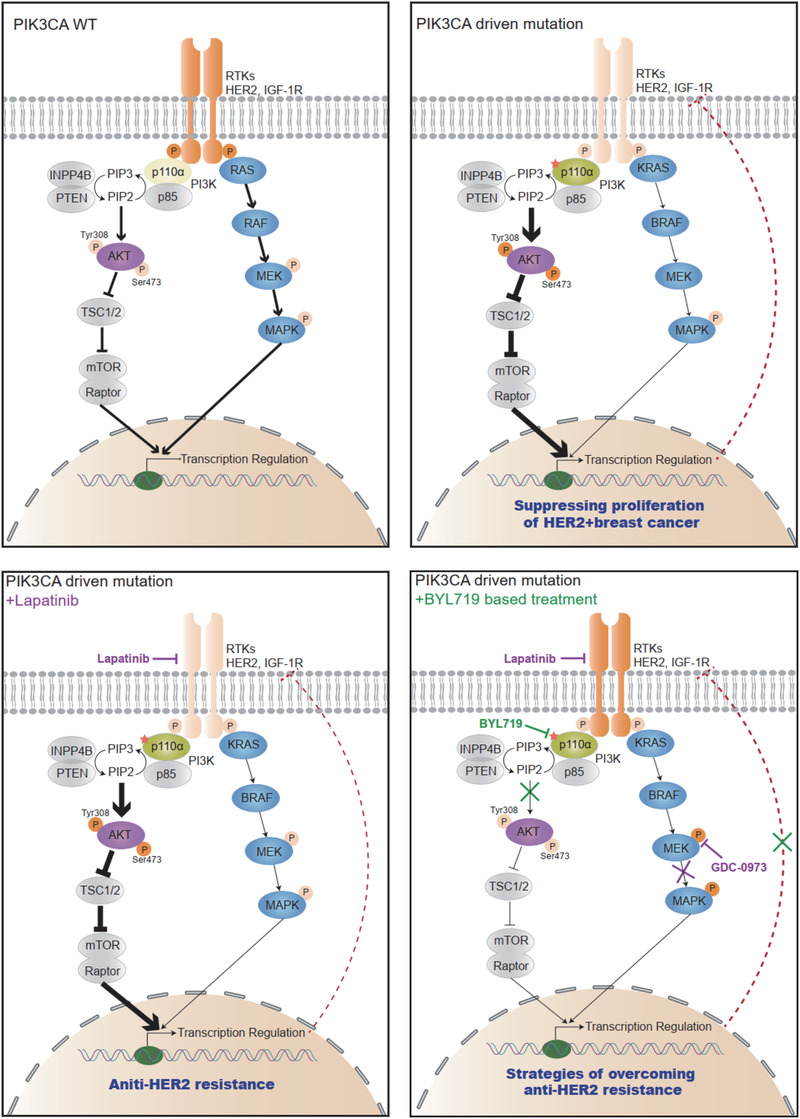
A diagram showing the adaptive strategies targeting mutated‐PI3Kα and enriched‐HER2 in breast cancer progression. *PIK3CA* functional mutations suppress the expression and activation of HER2 and other RTKs through a negative feedback loop, thus acting as a protective factor for treatment‐naïve HER2+ breast cancer. Meanwhile, mutated‐PI3Kα confers anti‐HER2 resistance for patients receiving continuous trastuzumab‐based neoadjuvant/adjuvant treatment. The tumours harbouring *PIK3CA* functional mutations are sensitive to the PI3K‐specific inhibitor BYL719, but the therapeutic effects will be interfered with rescued RTKs activation by abolishing the negative feedback. The combination of BYL719 and lapatinib shows a promising synergetic efficacy to target mutated‐PI3Kα and enriched‐HER2 simultaneously, offering a therapeutic potential to address multiple pathogens and improve outcomes for advanced HER2+ patients

Although patients with *PIK3CA* mutations had a lower pCR or ORR in the locally advanced and advanced cohorts, there were no significant correlation with distant OS, which was consistent with the NSABP31 trial.^33^ Both *PIK3CA* wild‐type and mutated patients benefited from adjuvant anti‐HER2 targeted therapy, and the FinHER trial^34^ even showed that the *PIK3CA* mutant cohort achieved a better survival outcome within the first 3 years. Similarly, in the Cancer Genome Atlas, the OS of the *PIK3CA*‐mutant HER2+ tumours got a better trend than that of the *PIK3CA*‐WT tumours, although there was no statistically significant (Broad Institute). We revealed the biological mechanism for this clinical observation and found the gain‐of‐function *PIK3CA* mutations mediated the inhibition of upstream signalling, which simultaneously affected the cross‐reactivity of RTK signalling and attenuated the activation of the MAPK pathway, which was consistent with preclinical evaluation of Serra and Elster et al.^35^, ^36^ Therefore, tumours carrying *PIK3CA* mutations grew slower than wild‐type tumours both in vivo and in vitro, which might be a unique phenotype for HER2+ breast cancer (Figure 7).

Some studies also hypothesised that *PIK3CA* mutations may play a pivotal role in tumour initiation and malignant transformation. Hyun et al. reported that the driven mutation was expected to have a relatively high VAF due to its early onset and selective benefit during carcinogenesis.^37^ Therefore, we argued that *PIK3CA* mutation might be a protective factor associated with improved outcome for treatment‐naïve HER2+ patients; while, with the extension of treatment time, *PIK3CA* wild‐type cells were killed by chemotherapeutic drugs, and the proportion of mutant tumour cells became the dominant clones, which played an important role in anti‐HER2 resistance with the prolongation of treatment endurance. We then proposed a combination strategy of lapatinib and BYL719 to reverse anti‐HER2 resistance by blocking amplified *HER2* and continuously activating *PIK3CA* simultaneously (Figure 7). A phase I study showed that the combination strategy of BYL719 and T‐DM1 was tolerable and efficacious to overcome anti‐HER2 resistance.^38^ Therefore, we proposed an individualised treatment plan based on different anti‐HER2 stages that PI3Kα and HER2 should be concomitantly inhibited when exposing to continuous anti‐HER2 treatment, while anti‐HER2 treatment‐naïve patients would not be necessary to receive targeted PI3Kα treatment. Additionally, patients who have received long‐term anti‐HER2 treatment and acquired secondary resistance to PI3K inhibitors could have a try on MAPK inhibitors to block compensatory MAPK signalling.

Collectively, our clinical sequencing program constructed one of the largest prospective breast cancer datasets to correlate the genomic characteristics and clinic‐pathological information. Although a certain percentage of patients have not achieved a complete pathological response and survival outcome, this FUSCC breast cancer dataset still provides powerful evidences to uncover the changing roles of *PIK3CA* mutations in treatment‐naïve or early‐stage patients and advanced‐stage HER2+ patients. The effective inhibition of both mutated‐PI3Kα and enriched‐HER2 offers therapeutic potential to address multiple detrimental factors to improve outcomes for advanced HER2+ breast cancer patients; meanwhile, anti‐HER2 treatment‐naïve patients would not be necessary to receive PI3Kα‐targeted treatment. Moreover, our study emphasises that the accurate annotation of genomic alterations and their corresponding treatment should vary with the different stages of tumour progression and their molecular characteristics.

## CONFLICT OF INTEREST

The authors have declared no conflicts of interest.

## Supporting information

Supplement information
