# Supplementary material for: Large‐scale genomic sequencing reveals adaptive opportunity of targeting mutated‐PI3Kα in early and advanced HER2‐positive breast cancer
Source: Clin Transl Med. 2021 Nov 4;11(11):e589. doi: 10.1002/ctm2.589 (PMC8567053; doi:10.1002/ctm2.589)
Supplement: Supplementary file 1 — Supplement information [file CTM2-11-e589-s001.docx]

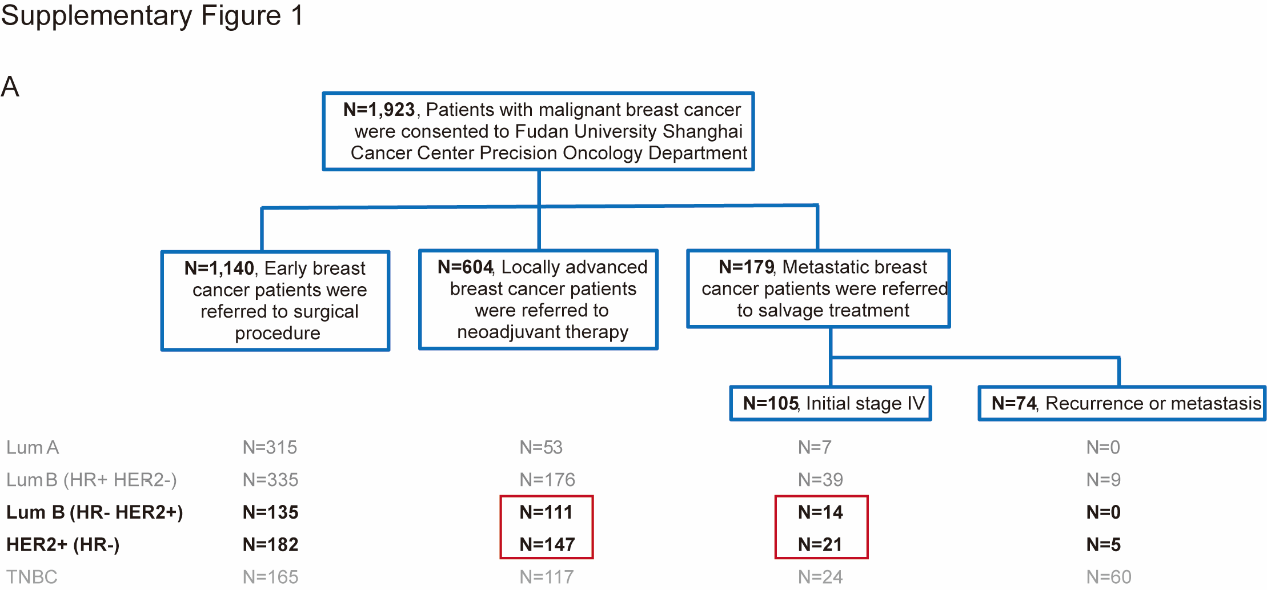


**Supplementary Fig. 1 The baseline information of FUSCC breast cancer cohort.**

**(A)** The clinical information and pathological subtype distribution of patients in the FUSCC-BC cohort.

**
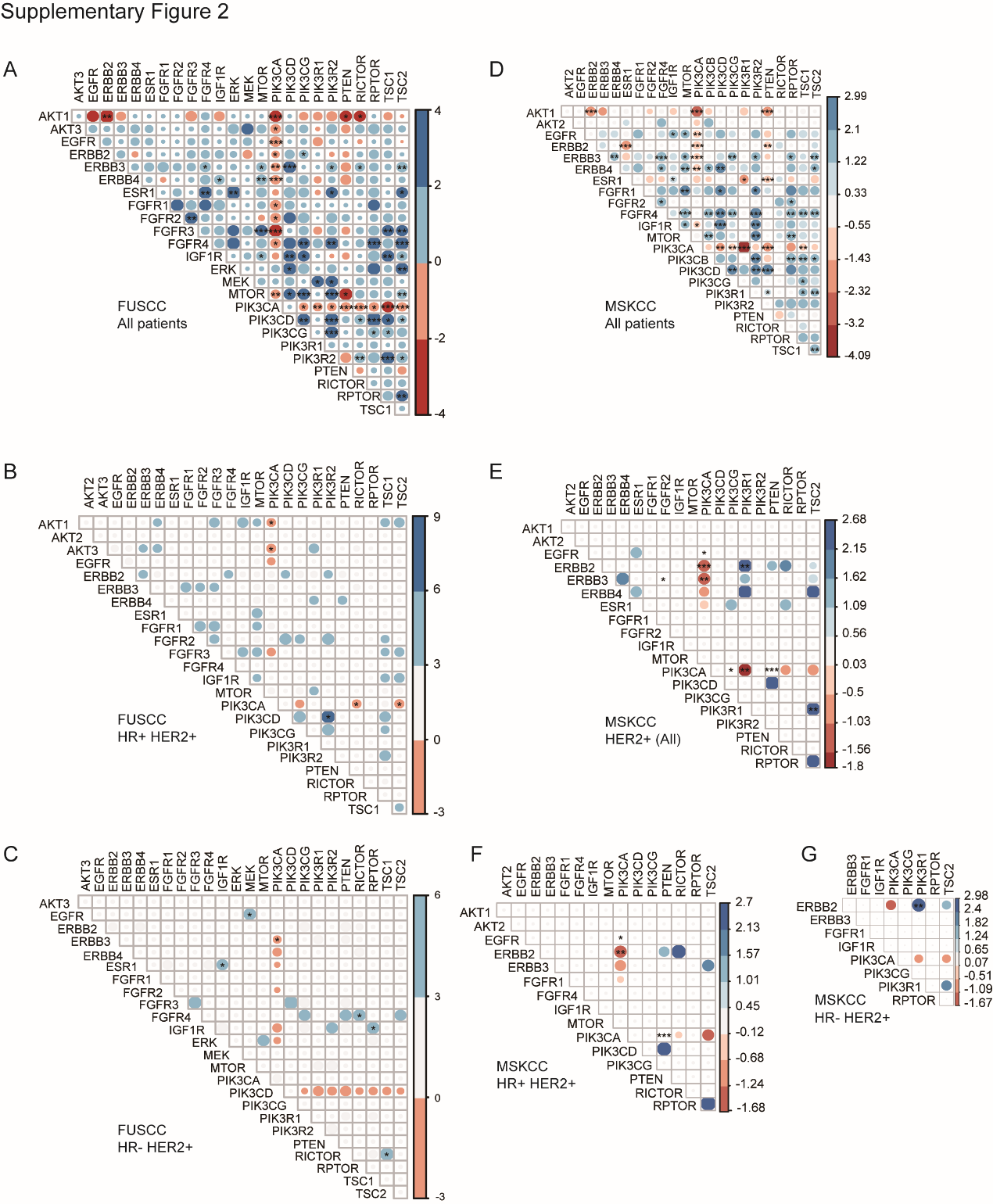
**

**Supplementary Fig. 2 Co-pairs relationship of *RTK-PI3K-MAPK* mutations in the FUSCC and MSKCC cohorts.**

Mutual cooccurrence (blue) and mutual exclusivity (red) of gene mutations among RTK-related pathways in the FUSCC **(A)**, HR+HER2+ **(B)** and HR-HER2+ cohorts **(C)**.

Mutual cooccurrence (blue) and mutual exclusivity (red) of gene mutations among RTK-related pathways in the MSKCC **(D)**, HER2+ (All) **(E)**, HR+HER2+ **(F)** and HR-HER2+ cohorts **(G)**. Scale bar: value of log10 ratio of odds ratio (OR); value scaled with color intensity. **p* < 0.05, ***p* < 0.01, ****p* < 0.001.

**Supplementary Fig.**
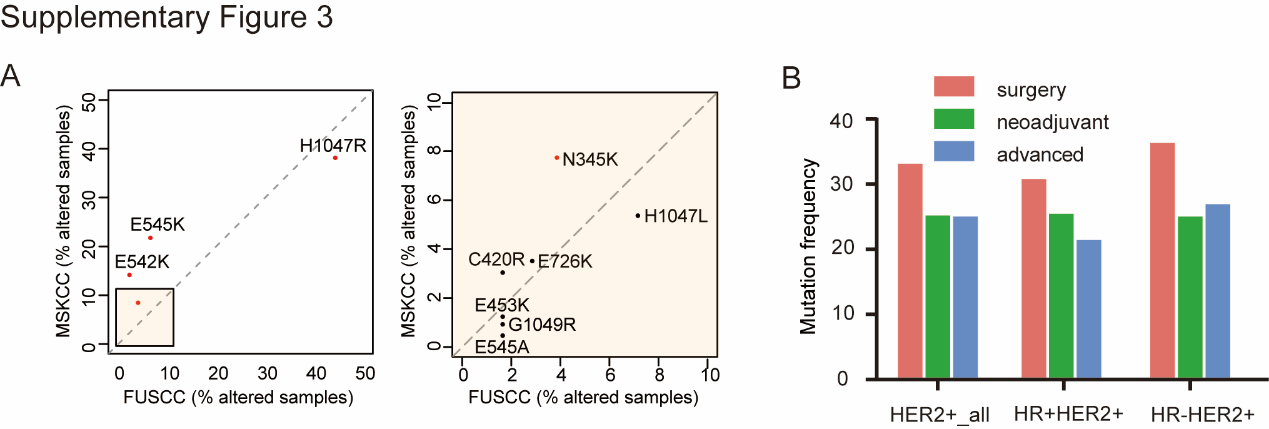
**3 Characteristics of *PIK3CA* genomic variants.**

1. Scatter plots of the prevalence of *PIK3CA* mutation spots in all breast cancer samples from FUSCC on the x-axis and from MSKCC on the y-axis. FDR < 0.05 (red plots), FDR > 0.05 (black plots).
2. The frequency of *PIK3CA* mutations in HER2-positive breast cancer patients referred to early breast cancer, locally advanced and advanced cohort.


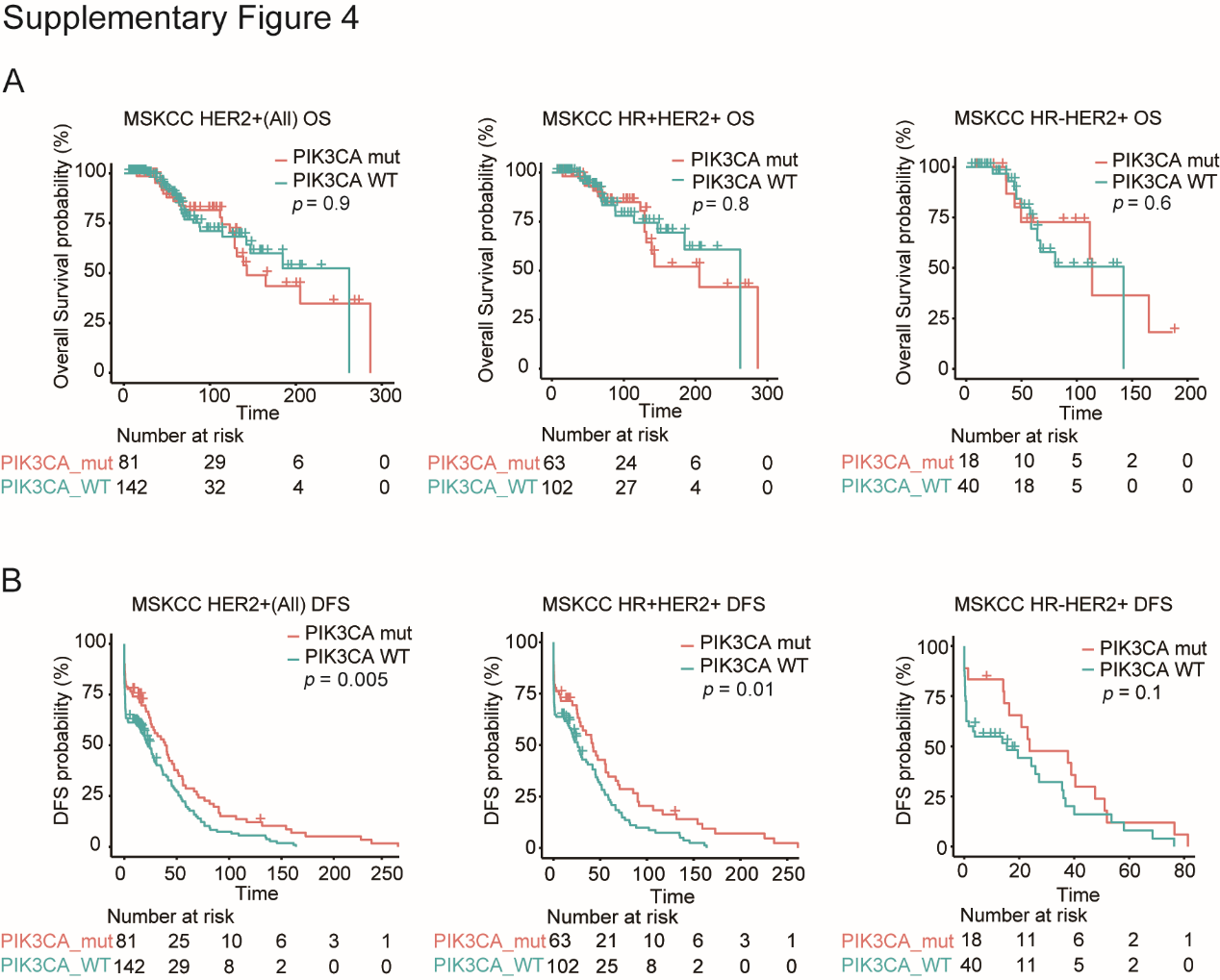


**Supplementary Fig. 4 Survival outcome of patients with mutant and wild-type *PIK3CA* in the MSKCC cohort.**

Cumulative OS **(A)** and DFS **(B)** curves of patients with WT and mutant *PIK3CA*. Data are shown for the HER2+ (All), HR+HER2+, HR-HER2+ breast cancer cohorts.

OR, overall response; CR, complete response; PR, partial response; PD, progressive disease.

**
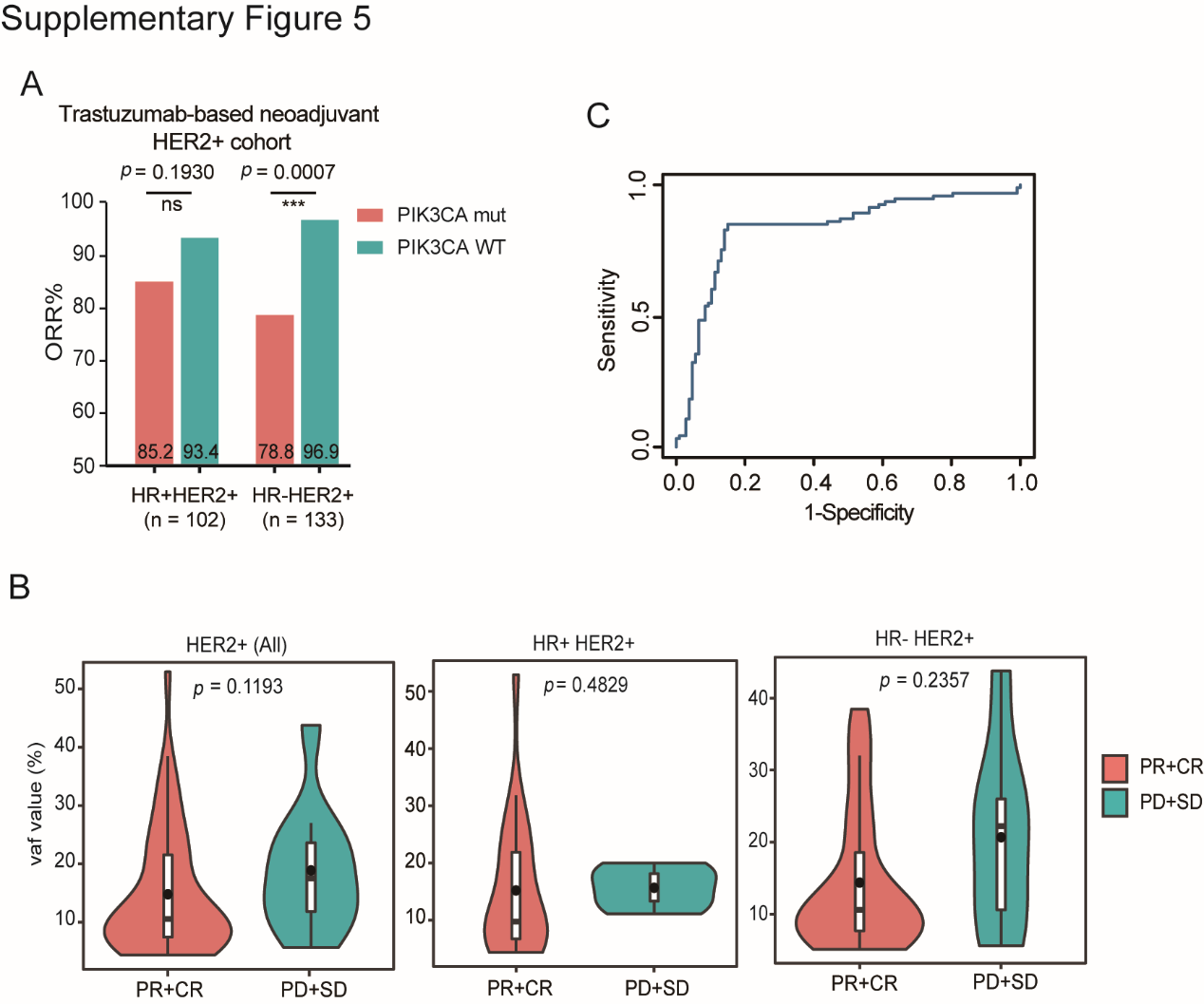
**

**Supplementary Fig. 5 Clinical significance of *PIK3CA* mutations.**

1. Objective response rate according to *PIK3CA* mutation status in the HR+HER2- and HR+HER2+ subtype using a fisher’s exact test of association**.**
2. Comparison of VAF distribution between the PR+CR and PD+SD groups in HER2+ (all), HR+HER2+, HR-HER2+ breast cancer patients with *PIK3CA* mutations.
3. ROC curve plots the true positive rate (sensitivity) on the vertical axis against the false-positive rate (1-specificity) on the horizontal axis. The cut off value VAF = 12.23% was determined at the point of [sensitivity-(1-specificity)]_max_.

**
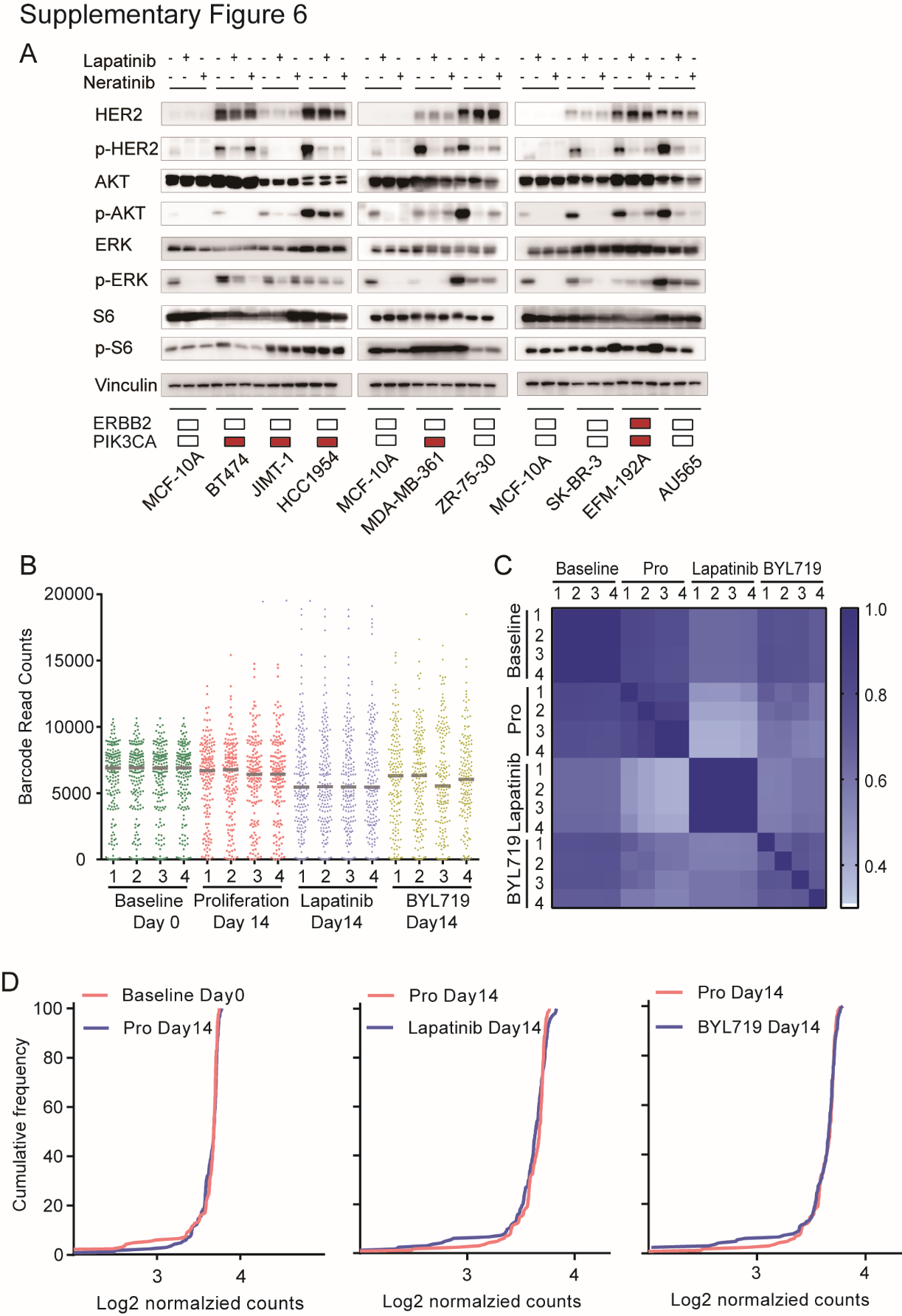
**

**Supplementary Fig. 6 Her2-positive cell line selection and quality control of library screening.**

**(A)** Suitable HER2+ cell line, without *PIK3CA* and *ERBB* family functional mutations, expose regular HER2-PI3K-MAPK pathway activation.

**(B)** Scatter plots of the *PIK3CA* mutation library screen.

**(C)** Pearson correlation coefficient of the normalized read counts between three different treatment conditions and biological replicates.

**(D)** Cumulative frequency of *PIK3CA* mutations in the proliferation assays on day 0 and day 14; lapatinib and BYL719 response assays on treatment day 14 and proliferation day 14, eliminating interference from proliferation.

**
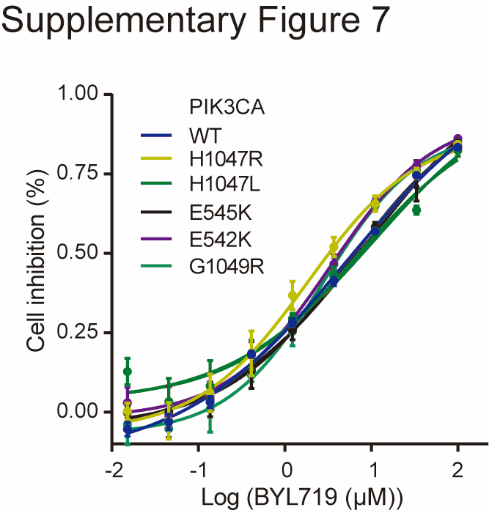
**

**Supplementary Fig. 7 HER2+ cells carrying *PIK3CA* driven mutations are sensitive to the PI3Kα inhibitor BYL719.**

*PIK3CA-*WT and *PIK3CA*-mutated SK-BR-3 breast cancer cells responded to BYL719 according to a CCK-8 assay. IC_50_ values were calculated after drug treatment for 5 days.

**
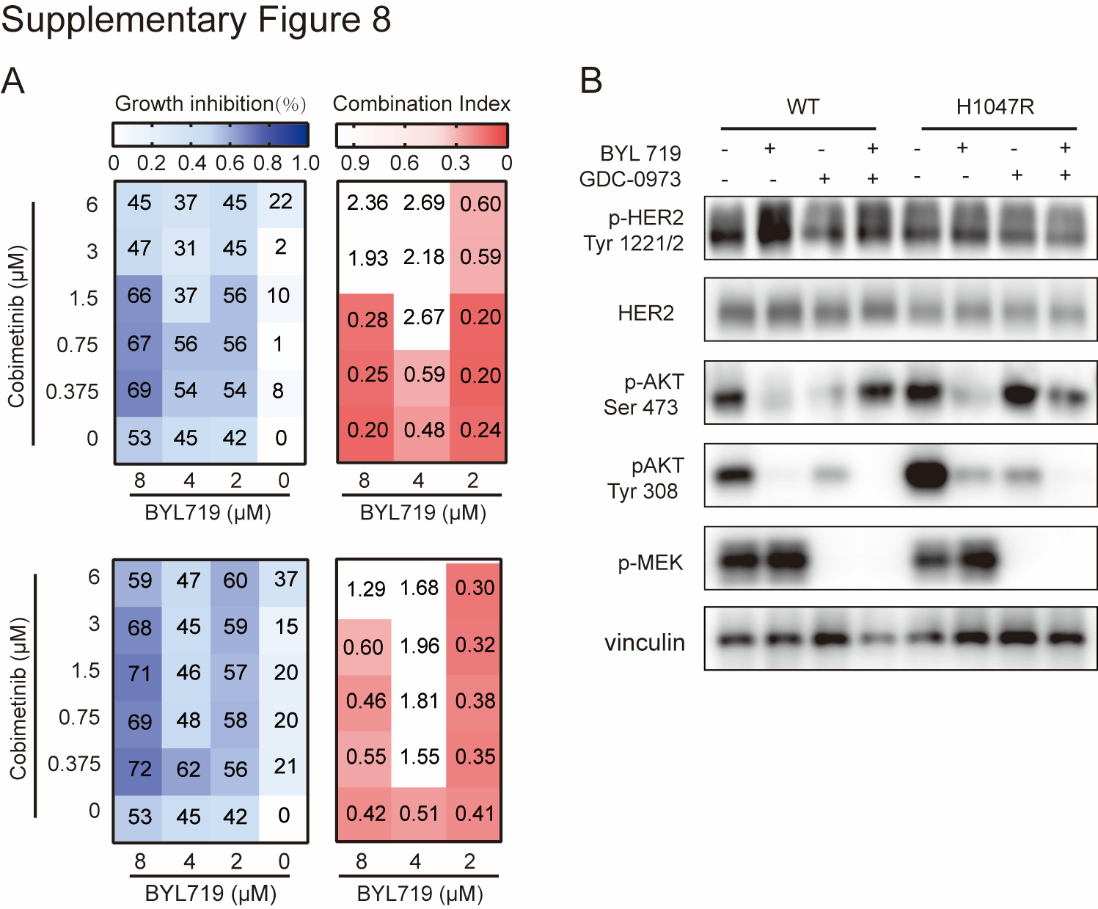
**

**Supplementary Fig. 8 BYL719 in combination with GDC-0973 synergizes at inhibiting cell proliferation of *PIK3CA*-mutant breast cancer.**

**(A)** *PIK3CA*^WT^and *PIK3CA*^H1047R^ cells were treated with BYL719, GDC-0973 and their combination as indicated. The percentage inhibition (left) and CI (right) at each concentration of the drugs are presented. Each score represents data from three independent experiments.

**(B)** Immunoblot analysis of *PIK3CA*^WT^, *PIK3CA*^H1047R^, and *PIK3CA*^E545K^ cells treated with BYL719 (2 𝜇M), GDC-0973 (750 nM) or their combination.

**
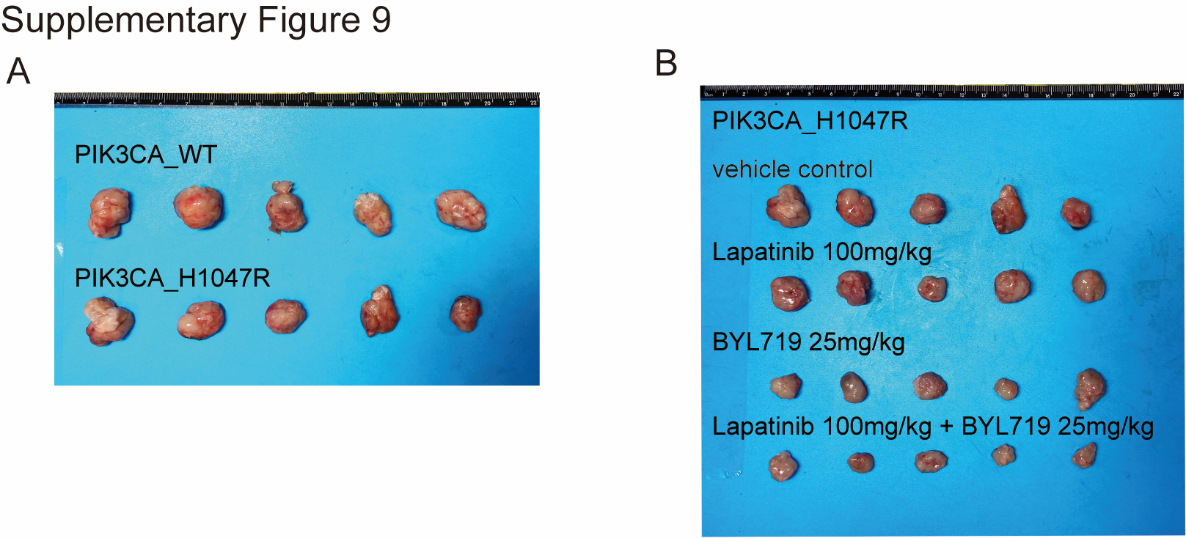
**

**Supplementary** **Fig. 9 Photographs of xenograft tumors from NOD/SCID mice injected with MCF-10CA1a-HER2-overexpressing cells transfected with *PIK3CA*^WT^ and *PIK3CA*^H1047R^.**

**(A)** Photographs of xenograft tumors carrying *PIK3CA*^WT^ and *PIK3CA*^H1047R^ from mice bearing for one month (n = 5).

**(B)** Images of four treatment groups of xenograft tumors with *PIK3CA*^H1047R^: vehicle control, lapatinib (100 mg/kg), BYL719 (25 mg/kg) and the combination group of lapatinib and BYL719 three weeks after administration.

| **1-100** | ABCB1 | CDC25B | EWSR1 | HSPA4 | MAP4K5 | NTRK1 | PRKX | SMARCB1 | ABL1 | CDC25C |
| --- | --- | --- | --- | --- | --- | --- | --- | --- | --- | --- |
|  | EXO1 | IDH1 | MAP7 | NTRK2 | PRMT2 | SMO | AFF2 | CDH1 | FAAH | IDH2 |
|  | MAPK1 | NTRK3 | PTCH1 | SMYD3 | AKAP3 | CDH16 | FAAP20 | IGF1 | MAPK10 | OGG1 |
|  | PTEN | SOCS2 | AKT1 | CDK16 | FAM47C | IGF1R | MAPK13 | SLC19A3 | PTGS2 | SOX9 |
|  | AKT2 | CDK2 | FAN1 | IKBKB | MAPK14 | OR2G3 | PTK2 | SPEN | AKT3 | CDK4 |
|  | FANCA | IL15RA | MAPK3 | OR2L2 | PTK2B | SRC | ALDH1A1 | CDK6 | FANCC | IL2 |
|  | MAPK4 | OR6A2 | PTK6 | SRMS | ALK | CDKN1B | FANCD2 | IL7R | MAPK6 | PAK1 |
|  | PTPN22 | SSTR5 | ALOX5 | CDKN2A | FANCE | INSR | MAPK7 | PAK2 | PTPRD | ST8SIA4 |
|  | ANKRD11 | CDKN2B | FANCF | INSRR | MAPRE2 | HSP90B1 | PTPRN2 | STAG2 | APC | CDC25A |
|  | FANCG | IRAK1 | MAPRE3 | PALB2 | STAT1 | MAP4K4 | APP | CHEK1 | IRF4 | ESR1 |

**Supplementary Table 1** FUSCC breast cancer sequencing panel: 484-gene list.

| **101-200** | MAPT | PALLD | RAB25 | STAT5A | ARAF | CHEK2 | FANCL | IRS1 | MAST2 | PARP1 |
| --- | --- | --- | --- | --- | --- | --- | --- | --- | --- | --- |
|  | RAC1 | STAT5B | ARID1A | CHML | FANCM | IRS2 | MCL1 | PARP2 | RAC2 | STK11 |
|  | ARID1B | CHRNA4 | FASN | ITCH | MDM2 | PBRM1 | RAD18 | SYK | ASXL1 | CHUK |
|  | FBXW7 | ITGB3 | MDM4 | PCID2 | RAD50 | TBL1XR1 | ATM | CIC | FER | ITGB4 |
|  | MECOM | PDE4B | RAD51 | TBX3 | ATN1 | PRKDC | FES | ITK | MECP2 | PDE4C |
|  | RAD51B | TEC | ATP2B2 | NR3C1 | FGFR1 | JAK1 | MED23 | PDE4D | RAD51C | TEK |
|  | ATR | CNR1 | FGFR2 | JAK2 | MEN1 | PDGFB | RAD51D | TEP1 | ATRX | COL1A1 |
|  | FGFR3 | JAK3 | MERTK | PDGFRA | RAD52 | TERT | AURKA | COL2A1 | FGFR4 | JUN |
|  | MET | PDGFRB | RAD54L | TET2 | AURKC | CREBBP | FGR | KAT6A | MICA | PEG10 |
|  | RAF1 | TGFBR1 | AXIN1 | NRAS | FH | KAT8 | MIEN1 | PFKFB3 | RASAL1 | TKTL1 |

| **201-300** | AXL | CRK | FLT1 | KCNB2 | MLH1 | PGR | RB1 | TLR4 | BAK1 | CSF1R |
| --- | --- | --- | --- | --- | --- | --- | --- | --- | --- | --- |
|  | FLT3 | KCNJ12 | MLLT4 | PHF6 | RBM17 | TLR9 | BARD1 | CSF3R | FLT4 | KCNJ14 |
|  | MMP1 | PHKA2 | SMARCA4 | TNFRSF11A | BAX | CSNK1E | FOXA1 | MAP4K3 | MMP14 | PI4K2A |
|  | REL | TNK2 | BCL2 | CSNK2A1 | FOXO3 | KCNJ3 | MMP2 | PI4KB | RET | TNKS2 |
|  | BCL2A1 | CTCF | FOXP1 | KCNJ5 | MMP3 | PIK3C3 | RFC1 | TOP1 | BCL2L2 | CTCFL |
|  | FOXQ1 | ZBP1 | MMP9 | PIK3CA | RGMB | TOP2A | BCOR | SMAD4 | ZHX2 | KCNJ9 |
|  | MRE11A | PIK3CB | RHOA | TOP2B | BCR | CUX1 | FSIP1 | KDM1B | MSH2 | PIK3CD |
|  | RICTOR | TOP3A | BIRC2 | CYP19A1 | FYN | KDM4A | MSH6 | PIK3CG | RIPK1 | TOP3B |
|  | BIRC3 | CYP2C8 | GAB2 | KDM5A | MST1R | PIK3R1 | ROCK1 | TP53 | BLK | CYP2C9 |
|  | GATA3 | KDM6A | MTAP | PIK3R2 | ROCK2 | TPMT | BLM | CYP3A4 | GHRL | KDR |

| **301-400** | MTHFR | PIKFYVE | ROS1 | TPRX1 | BMP7 | DCAF4L2 | GLIS3 | KIF11 | MTOR | PIM1 |
| --- | --- | --- | --- | --- | --- | --- | --- | --- | --- | --- |
|  | RPA1 | TRIM47 | BMPR1A | DEK | GNAS | KIF26B | MUTYH | PIWIL1 | RPGR | TRIM65 |
|  | BMX | DGKG | GOLPH3L | KIT | MYB | PKD1 | RPS6KA1 | CCNE1 | BRAF | DNMT1 |
|  | GPR32 | KLLN | MYC | PLCG1 | RPS6KA3 | TRPS1 | BRCA1 | DNMT3A | GPS2 | KMT2B |
|  | NBN | PLK2 | RPS6KA5 | TSC1 | BRCA2 | DNMT3B | GRB7 | KMT2D | NCOA1 | PLK3 |
|  | RPS6KB1 | TSC2 | BRIP1 | DUSP7 | GRIN2A | KRAS | NCOR1 | PMS2 | RPTOR | TTK |
|  | HSP90AB1 | DYRK1A | GSK3A | LCK | NEK2 | PNPLA3 | RRM1 | TUSC5 | BUB1B | ECT2L |
|  | GSK3B | LPL | NF1 | POLQ | RRM2 | TYK2 | CACNA1D | EGFR | HDAC1 | LYN |
|  | NF2 | ERBB4 | RUNX1 | TYRO3 | CAMK2G | EHMT2 | HDAC2 | MAP1A | NFIB | PPARA |
|  | RYR2 | UNC13D | CASP8 | EPAS1 | HDAC4 | MAP1B | NFKB1 | PPARG | CCND3 | USH2A |

| **401-484** | HRAS | EPCAM | HDAC5 | MAP2 | NFKB2 | PRDM1 | S1PR2 | USP9X | CBFB | EPHA1 |
| --- | --- | --- | --- | --- | --- | --- | --- | --- | --- | --- |
|  | HDAC6 | MAP2K1 | NFKBIA | PREX2 | SETD2 | VHL | CBL | EPHA2 | HDAC7 | MAP2K2 |
|  | NOS1 | PRKAA1 | SF3B1 | VPS4B | CBLB | EPHA3 | HDAC9 | MAP2K4 | NOS2 | NR2F2 |
|  | SGK1 | WEE1 | CCL1 | EPHB1 | HIF1A | MAP3K1 | NOS3 | PRKACB | SGK2 | WNK3 |
|  | CCL2 | EPHB2 | PRKCQ | MAP3K13 | NOTCH1 | PRKCA | SHC1 | WNT7A | CCL20 | EPHB4 |
|  | MAP3K7 | MAP3K2 | NOTCH2 | PRKCB | SHH | XBP1 | CCL7 | EPSTI1 | HK2 | MAP3K4 |
|  | NOTCH3 | PRKCD | SIK2 | XDH | CCL8 | ERBB2 | HK3 | MAP3K5 | NR1H2 | PRKCE |
|  | SIRT1 | YES1 | CCND1 | ERBB3 | HMGCR | MAP3K6 | NR1H4 | PRKCG | SIRT7 | ZAP70 |
|  | ZFP36L1 | MAP4 | PRKCZ | SLC5A1 |  |  |  |  |  |  |

**Supplementary Table 2** Clinic-pathological parameters of the FUSCC-BC cohort.

| **Variables** | **Number of** | ***PIK3CA*-mutant** | | ***P* value** |
| --- | --- | --- | --- | --- |
|  | **patients (%)** | **Positive (%)** | **Negative (%)** |  |
| **Total** | 1923 | 631(32.8) | 1292(67.2) |  |
| **Age** |  |  |  | ***p* < 0.0001** |
| ≤40 | 222 (11.5) | 64 (3.3) | 158 (8.2) |  |
| 40-60 | 955 (49.7) | 378(19.7) | 577 (30) |  |
| >60 | 746 (38.8) | 189 (9.8) | 557 (29) |  |
| **Menopause** |  |  |  | ***p* = 0.0043** |
| Yes | 947 (49.2) | 281 (14.6) | 666 (34.6) |  |
| No | 978 (50.8) | 350 (18.2) | 628 (32.7) |  |
| **cT** |  |  |  | ***p* = 0.0189** |
| Tis (DCIS) | 17 (0.9) | 11 (0.6) | 6 (0.3) |  |
| 1 | 638 (33.2) | 228 (11.9) | 410 (21.3) |  |
| 2 | 910 (47.3) | 293 (15.2) | 617 (32.1) |  |
| 3 | 174 (9.0) | 49 (2.5) | 125 (6.5) |  |
| 4 | 67 (3.5) | 22 (1.1) | 45 (2.4) |  |
| NA | 117 (6.1) | 34 (1.8) | 83 (4.3) |  |
| **cN** |  |  |  | ***p* = 0.0754** |
| 0 | 775 (40.3) | 273 (14.2) | 502 (26.1) |  |
| 1 | 443 (23.0) | 151 (7.9) | 292 (15.1) |  |
| 2 | 383 (19.9) | 116 (6.0） | 267 (13.9) |  |
| 3 | 209 (10.9) | 56 (2.9) | 153 (8.0) |  |
| NA | 113 (5.9) | 34 (1.8) | 79 (4.1) |  |
| **cM** |  |  |  | *p* = 0.4660 |
| 0 | 1729 (89.9) | 572 (29.7) | 1157 (60.2) |  |
| 1 | 111 (5.8) | 33 (1.7) | 78 (4.1) |  |
| NA | 83 (4.3) | 25 (1.3) | 58 (3.0) |  |
| **cStage** |  |  |  | ***p*=0.0026** |
| 0 | 2 (0.1) | 2 (0.1) | 0 (0) |  |
| 1 | 421 (21.9) | 159 (8.3) | 262 (13.6) |  |
| 2 | 797 (41.4) | 273 (14.2) | 524 (27.2) |  |
| 3 | 511 (26.6) | 141 (7.3) | 370 (19.2) |  |
| 4 | 109 (5.7) | 0 (0) | 77 (4.0) |  |
| NA | 83 (4.3) | 25 (1.3) | 58 (3.0) |  |
| **ER** |  |  |  | ***p* = 0.0003** |
| Positive | 1208 (62.8) | 429 (22.3) | 779 (40.5) |  |
| Negative | 702 (36.5) | 193 (10.0) | 509 (26.5) |  |
| NA | 13 (0.7) | 8 (0.4) | 5 (0.27) |  |
| **PR** |  |  |  | ***p* < 0.0001** |
| Positive | 1003 (52.2) | 374 (19.4) | 629 (32.7) |  |
| Negative | 907 (47.2) | 248 (12.9) | 659 (34.3) |  |
| NA | 13 (0.6) | 8 (0.4) | 5 (0.27) |  |
| **HER2** |  |  |  | ***p* = 0.0078** |
| Positive | 613 (31.9) | 174 (9.0) | 439 (22.9) |  |
| Negative | 1284 (66.8) | 443 (23.0) | 841 (43.8) |  |
| NA | 26 (1.3) | 14 (0.7) | 12 (0.6) |  |

Abbreviations: cT, tumor size; cN, lymph nodes status; cM, metastasis; ER, estrogen receptor; PR, progesterone receptor; HER2, human epidermal growth factor receptor 2.

| **Supplementary Table 3:** Specific genes in RTK, PI3K and RAS-MAPK pathways. | | |
| --- | --- | --- |
| **PI3K** | **RTK** | **RAS-MAPK** |
| AKT1 | EGFR | KRAS |
| AKT2 | ERBB2 | HRAS |
| AKT3 | ERBB3 | NRAS |
| MTOR | ERBB4 | RAF1 |
| PIK3CA | PDGFRA | RAC1 |
| PIK3CB | PDGFRB | ARAF |
| PIK3R1 | MET | BRAF |
| PIK3R2 | FGFR1 | MAP2K1 |
| PTEN | FGFR2 | MAP2K2 |
| RICTOR | FGFR3 | MAPK1 |
| RPTOR | FGFR4 | MAPK3 |
| RPS6KB1 | IGF1R | RASAL1 |
| STK11 | NF1 | JAK2 |
| TSC1 | NTRK1 |  |
| TSC2 | NTRK2 |  |
|  | NTRK3 |  |
|  | RET |  |
|  | INSR |  |
|  | INSRR |  |
|  | IRS1 |  |
|  | IRS2 |  |
|  | ABL1 |  |
|  | FLT3 |  |
|  | ALK |  |
|  | ROS1 |  |
|  | KIT |  |
|  | SOS1 |  |
|  | GRB2 |  |
|  | CBL |  |
|  | CBLB |  |
|  | SHC1 |  |

| **Supplementary Table 4** Correlation of *PIK3CA* mutations and anti-HER2 treatment efficacy in the neoadjuvant settings. | | | |
| --- | --- | --- | --- |
| **(A) Trastuzumab-based neoadjuvant therapy** | | | |
| **HR+HER2+** | PR | PD | Total |
| *PIK3CA*_mut | 22 | 4 | 26 |
| *PIK3CA*_WT | 71 | 5 | 76 |
| Total | 95 | 9 | 102 |
| *p* = 0.1930 | |  |  |
|  |  |  |  |
| **HR-HER2+** | PR | PD | Total |
| *PIK3CA*_mut | 27 | 7 | 34 |
| *PIK3CA*_WT | 96 | 3 | 99 |
| Total | 121 | 10 | 133 |
| ***p* = 0.0007** | |  |  |
|  |  |  |  |
| **(B) VAF threshold (HR-HER2+)** | | | |
|  | VAF ≥ 12.23 | VAF < 12.23 | Total |
| PD+SD | 5 | 1 | 6 |
| PR+CR | 10 | 15 | 25 |
| Total | 15 | 16 | 31 |
| *p* = 0.0829 |  |  |  |

**Supplementary Table 5** List of *PIK3CA*-mutation library.

| **List of 119 *PIK3CA* mutation library** | | |
| --- | --- | --- |
| **Mutation ID** | **cDNA change** | **AA change** |
| 1 | a88g | p.M30V |
| 2 | g115a | p.E39K |
| 3 | a153t | p.K51N |
| 4 | a227g | p.E76G |
| 5 | g333t | p.K111N |
| 6 | a604g | p.N202D |
| 7 | t632g | p.I211S |
| 8 | a656g | p.Q219R |
| 9 | a1540g | p.S514G |
| 10 | a1546g | p.R516G |
| 11 | a2129g | p.E710G |
| 12 | g2176a | p.E726K |
| 13 | a2279t | p.Q760L |
| 14 | t1258c | p.C420R |
| 15 | t1492a | p.W498R |
| 16 | g1624a | p.E542K |
| 17 | g1633a | p.E545K |
| 18 | c1636a | p.Q546K |
| 19 | a3121g | p.K1041E |
| 20 | a3140g | p.H1047R |
| 21 | a93g | p.I31M |
| 22 | g113a | p.R38H |
| 23 | c178a | p.Q60K |
| 24 | g238a | p.E80K |
| 25 | g241a | p.E81K |
| 26 | g263a | p.R88Q |
| 27 | c311t | p.P104L |
| 28 | g323a | p.R108H |
| 29 | g328a | p.E110K |
| 30 | g353a | p.G118D |
| 31 | g1030a | p.V344M |
| 32 | a1033c | p.N345H |
| 33 | a1034c | p.N345T |
| 34 | a1034t | p.N345I |
| 35 | t1035g | p.N345K |
| 36 | a1049g | p.D350G |
| 37 | a1094t | p.E365V |
| 38 | c1097g | p.P366R |
| 39 | g1133t | p.C378F |
| 40 | g1193a | p.R398H |
| 41 | c1214t | p.S405F |
| 42 | g1252a | p.E418K |
| 43 | g1357a | p.E453K |
| 44 | c1371g | p.N457K |
| 45 | c1411g | p.P471A |
| 46 | a1490g | p.N497S |
| 47 | a1543g | p.N515D |
| 48 | t1568c | p.L523S |
| 49 | g1573a | p.E525K |
| 50 | t1592c | p.L531P |
| 51 | c1598t | p.A533V |
| 52 | g1612t | p.D538Y |
| 53 | c1616g | p.P539R |
| 54 | c1618t | p.L540F |
| 55 | g1624c | p.E542Q |
| 56 | g1624a_a1625g | p.E542R |
| 57 | a1625g | p.E542G |
| 58 | a1625t | p.E542V |
| 59 | g1633c | p.E545Q |
| 60 | a1634c | p.E545A |
| 61 | a1634g | p.E545G |
| 62 | g1635t | p.E545D |
| 63 | c1636g | p.Q546E |
| 64 | a1637c | p.Q546P |
| 65 | a1637g | p.Q546R |
| 66 | a1637t | p.Q546L |
| 67 | g1639a | p.E547K |
| 68 | g1658a | p.S553N |
| 69 | c1697t | p.P566L |
| 70 | a1700g | p.K567R |
| 71 | g1807c | p.D603H |
| 72 | t1840a | p.F614I |
| 73 | c1886g | p.S629C |
| 74 | t2017a | p.S673T |
| 75 | a2102c | p.H701P |
| 76 | g2119a | p.E707K |
| 77 | c2155g | p.L719V |
| 78 | c2296t | p.L766F |
| 79 | g2702t | p.C901F |
| 80 | c2727a | p.F909L |
| 81 | g2740a | p.G914R |
| 82 | a2816g | p.D939G |
| 83 | g2908a | p.E970K |
| 84 | c2965g | p.L989V |
| 85 | g3012a | p.M1004I |
| 86 | g3019c | p.G1007R |
| 87 | g3034a | p.E1012K |
| 88 | c3059t | p.A1020V |
| 89 | a3062g | p.Y1021C |
| 90 | g3068t | p.R1023L |
| 91 | c3074g | p.T1025S |
| 92 | c3074t | p.T1025I |
| 93 | g3085c | p.D1029H |
| 94 | c3104t | p.A1035V |
| 95 | g3109a | p.E1037K |
| 96 | a3118g | p.M1040V |
| 97 | g3120a | p.M1040I |
| 98 | a3127g | p.M1043V |
| 99 | g3129a | p.M1043I |
| 100 | a3130t | p.N1044Y |
| 101 | t3132g | p.N1044K |
| 102 | g3136a_a3138g | p.A1046T |
| 103 | c3137t | p.A1046V |
| 104 | c3139t | p.H1047Y |
| 105 | c3139a_a3140c | p.H1047T |
| 106 | a3143g | p.H1048R |
| 107 | a3143t | p.H1048L |
| 108 | g3146c | p.G1049A |
| 109 | a3154g | p.T1052A |
| 110 | c3155a | p.T1052K |
| 111 | a3184g | p.I1062V |
| 112 | a3194t | p.H1065L |
| 113 | c3197t | p.A1066V |
| 114 | a3140t | p.H1047L |
| 115 | t1395g | p.N465K |
| 116 | g1093a | p.E365K |
| 117 | a1474g | p.I492V |
| 118 | a1173g | p.I391M |
| 119 | g3145c | p.G1049R |

**Supplementary Table 6** Ranking of *PIK3CA* mutations induced proliferation.

| **Ranking** | **cDNA change** | **AA change** | **fold change (D14/D0)** |
| --- | --- | --- | --- |
| 1 | GFP | GFP | 1.65 |
| 2 | t1592c | p.L531P | 1.60 |
| 3 | c3139a_a3140c | p.H1047T | 1.57 |
| 4 | t632g | p.I211S | 1.49 |
| 5 | g1030a | p.V344M | 1.49 |
| 6 | GFP | GFP | 1.39 |
| 7 | a93g | p.I31M | 1.37 |
| 8 | c178a | p.Q60K | 1.36 |
| 9 | t1568c | p.L523S | 1.34 |
| 11 | g1624c | p.E542Q | 1.33 |
| 12 | c311t | p.P104L | 1.30 |
| 13 | a1543g | p.N515D | 1.25 |
| 14 | a604g | p.N202D | 1.21 |
| 15 | g115a | p.E39K | 1.21 |
| 16 | a1700g | p.K567R | 1.18 |
| 17 | c3137t | p.A1046V | 1.18 |
| 18 | WT | WT | 1.17 |
| 19 | a1173g | p.I391M | 1.16 |
| 20 | g3034a | p.E1012K | 1.16 |
| 22 | a3143t | p.H1048L | 1.16 |
| 24 | a2129g | p.E710G | 1.15 |
| 25 | g1193a | p.R398H | 1.14 |
| 26 | t1395g | p.N465K | 1.14 |
| 27 | c2296t | p.L766F | 1.14 |
| 28 | a227g | p.E76G | 1.14 |
| 30 | a153t | p.K51N | 1.14 |
| 31 | a1490g | p.N497S | 1.14 |
| 32 | c1598t | p.A533V | 1.13 |
| 33 | c1411g | p.P471A | 1.12 |
| 34 | g238a | p.E80K | 1.11 |
| 35 | WT | WT | 1.10 |
| 36 | g1357a | p.E453K | 1.10 |
| 37 | g3136a_a3138g | p.A1046T | 1.10 |
| 38 | a3121g | p.K1041E | 1.08 |
| 40 | a3154g | p.T1052A | 1.08 |
| 41 | a656g | p.Q219R | 1.08 |
| 42 | a3184g | p.I1062V | 1.07 |
| 43 | a3118g | p.M1040V | 1.06 |
| 44 | g1573a | p.E525K | 1.06 |
| 45 | c2965g | p.L989V | 1.05 |
| 46 | c1214t | p.S405F | 1.05 |
| 47 | g3085c | p.D1029H | 1.05 |
| 48 | g3012a | p.M1004I | 1.04 |
| 49 | a1033c | p.N345H | 1.03 |
| 50 | a3130t | p.N1044Y | 1.02 |
| 52 | c1097g | p.P366R | 0.99 |
| 53 | g1639a | p.E547K | 0.98 |
| 54 | a1049g | p.D350G | 0.98 |
| 55 | a1094t | p.E365V | 0.98 |
| 56 | g113a | p.R38H | 0.97 |
| 57 | g2740a | p.G914R | 0.97 |
| 58 | c1371g | p.N457K | 0.97 |
| 59 | g3129a | p.M1043I | 0.96 |
| 60 | t1492a | p.W498R | 0.96 |
| 61 | a1474g | p.I492V | 0.96 |
| 62 | g323a | p.R108H | 0.96 |
| 63 | c1636g | p.Q546E | 0.96 |
| 64 | c3074t | p.T1025I | 0.95 |
| 65 | c1697t | p.P566L | 0.95 |
| 66 | g1807c | p.D603H | 0.95 |
| 67 | g3109a | p.E1037K | 0.94 |
| 68 | g263a | p.R88Q | 0.94 |
| 69 | g2119a | p.E707K | 0.93 |
| 70 | c3104t | p.A1035V | 0.93 |
| 71 | g328a | p.E110K | 0.90 |
| 72 | a2279t | p.Q760L | 0.88 |
| 73 | c2727a | p.F909L | 0.87 |
| 74 | t3132g | p.N1044K | 0.87 |
| 75 | g353a | p.G118D | 0.86 |
| 76 | g241a | p.E81K | 0.86 |
| 77 | a1546g | p.R516G | 0.85 |
| 78 | g1633a | p.E545K | 0.84 |
| 79 | g1252a | p.E418K | 0.83 |
| 80 | g1624a | p.E542K | 0.83 |
| 81 | c3197t | p.A1066V | 0.82 |
| 82 | c1616g | p.P539R | 0.81 |
| 83 | a3127g | p.M1043V | 0.81 |
| 84 | g1633c | p.E545Q | 0.80 |
| 85 | a88g | p.M30V | 0.79 |
| 86 | g3120a | p.M1040I | 0.79 |
| 87 | g1658a | p.S553N | 0.79 |
| 88 | c3139t | p.H1047Y | 0.79 |
| 89 | a1034t | p.N345I | 0.78 |
| 90 | g3146c | p.G1049A | 0.77 |
| 91 | t1840a | p.F614I | 0.77 |
| 92 | a3194t | p.H1065L | 0.77 |
| 93 | g1093a | p.E365K | 0.77 |
| 94 | g1612t | p.D538Y | 0.77 |
| 95 | a1637t | p.Q546L | 0.76 |
| 96 | c3074g | p.T1025S | 0.76 |
| 97 | g333t | p.K111N | 0.75 |
| 98 | c2155g | p.L719V | 0.74 |
| 99 | g1133t | p.C378F | 0.74 |
| 100 | a1625g | p.E542G | 0.73 |
| 101 | c3059t | p.A1020V | 0.73 |
| 103 | t2017a | p.S673T | 0.70 |
| 104 | c1618t | p.L540F | 0.68 |
| 105 | a1637c | p.Q546P | 0.68 |
| 106 | g2908a | p.E970K | 0.68 |
| 107 | g1624a_a1625g | p.E542R | 0.68 |
| 108 | a1625t | p.E542V | 0.66 |
| 109 | g1635t | p.E545D | 0.66 |
| 110 | a2816g | p.D939G | 0.65 |
| 111 | a3143g | p.H1048R | 0.65 |
| 112 | g2176a | p.E726K | 0.64 |
| 113 | a3140t | p.H1047L | 0.62 |
| 114 | t1035g | p.N345K | 0.62 |
| 115 | g3019c | p.G1007R | 0.59 |
| 116 | t1258c | p.C420R | 0.59 |
| 117 | a1637g | p.Q546R | 0.58 |
| 118 | c3155a | p.T1052K | 0.57 |
| 119 | g2702t | p.C901F | 0.57 |
| 121 | a1634g | p.E545G | 0.50 |
| 122 | a3062g | p.Y1021C | 0.48 |
| 123 | a1634c | p.E545A | 0.46 |
| 124 | g3145c | p.G1049R | 0.46 |
| 125 | a3140g | p.H1047R | 0.32 |
| 126 | c1886g | p.S629C | 0.31 |

**Supplementary Table 7**

Ranking of *PIK3CA* mutations induced tyrosine kinase inhibitor lapatinib response.

| **Ranking** | **cDNA change** | **AA change** | **fold change (Lapatinib D14/D14)** |
| --- | --- | --- | --- |
| 1 | a3140g | p.H1047R | 1.68 |
| 2 | a1634g | p.E545G | 1.64 |
| 3 | g3145c | p.G1049R | 1.60 |
| 4 | a3062g | p.Y1021C | 1.53 |
| 5 | g1624a_a1625g | p.E542R | 1.47 |
| 6 | a1634c | p.E545A | 1.45 |
| 7 | g1624a | p.E542K | 1.42 |
| 8 | a1637c | p.Q546P | 1.41 |
| 9 | t1035g | p.N345K | 1.39 |
| 10 | c1697t | p.P566L | 1.39 |
| 11 | a1034t | p.N345I | 1.37 |
| 12 | a1637g | p.Q546R | 1.33 |
| 13 | a3140t | p.H1047L | 1.32 |
| 14 | g2908a | p.E970K | 1.31 |
| 15 | t3132g | p.N1044K | 1.28 |
| 16 | g1635t | p.E545D | 1.27 |
| 17 | a3143t | p.H1048L | 1.24 |
| 18 | g3120a | p.M1040I | 1.22 |
| 19 | a1094t | p.E365V | 1.21 |
| 20 | c1618t | p.L540F | 1.20 |
| 21 | g1252a | p.E418K | 1.19 |
| 22 | g263a | p.R88Q | 1.19 |
| 23 | c3155a | p.T1052K | 1.18 |
| 24 | a1625g | p.E542G | 1.18 |
| 25 | g1633a | p.E545K | 1.17 |
| 26 | g241a | p.E81K | 1.16 |
| 27 | a3194t | p.H1065L | 1.14 |
| 28 | a3127g | p.M1043V | 1.14 |
| 29 | a2816g | p.D939G | 1.14 |
| 30 | c3074g | p.T1025S | 1.13 |
| 31 | t1258c | p.C420R | 1.13 |
| 32 | a1474g | p.I492V | 1.12 |
| 33 | a1625t | p.E542V | 1.11 |
| 34 | g328a | p.E110K | 1.11 |
| 35 | g333t | p.K111N | 1.11 |
| 36 | c2727a | p.F909L | 1.09 |
| 37 | g1639a | p.E547K | 1.09 |
| 38 | a1173g | p.I391M | 1.08 |
| 39 | g353a | p.G118D | 1.07 |
| 40 | t632g | p.I211S | 1.07 |
| 41 | g2176a | p.E726K | 1.07 |
| 42 | g3085c | p.D1029H | 1.06 |
| 43 | a153t | p.K51N | 1.06 |
| 44 | g1093a | p.E365K | 1.04 |
| 45 | a3130t | p.N1044Y | 1.04 |
| 46 | c2965g | p.L989V | 1.03 |
| 47 | a2279t | p.Q760L | 1.02 |
| 48 | c1636g | p.Q546E | 1.02 |
| 49 | c2296t | p.L766F | 1.01 |
| 50 | g1633c | p.E545Q | 1.01 |
| 51 | a1637t | p.Q546L | 1.01 |
| 52 | c1616g | p.P539R | 1.00 |
| 53 | c3139t | p.H1047Y | 1.00 |
| 54 | a93g | p.I31M | 0.99 |
| 55 | c1598t | p.A533V | 0.99 |
| 56 | a1543g | p.N515D | 0.99 |
| 57 | g2702t | p.C901F | 0.99 |
| 58 | g2119a | p.E707K | 0.98 |
| 59 | c3197t | p.A1066V | 0.98 |
| 60 | a88g | p.M30V | 0.97 |
| 61 | a1033c | p.N345H | 0.97 |
| 62 | a2129g | p.E710G | 0.96 |
| 63 | c1097g | p.P366R | 0.95 |
| 64 | a1049g | p.D350G | 0.95 |
| 65 | a3143g | p.H1048R | 0.95 |
| 66 | g1612t | p.D538Y | 0.94 |
| 67 | t1395g | p.N465K | 0.94 |
| 68 | g2740a | p.G914R | 0.94 |
| 69 | g113a | p.R38H | 0.94 |
| 70 | c2155g | p.L719V | 0.93 |
| 71 | c1411g | p.P471A | 0.93 |
| 72 | g1624c | p.E542Q | 0.93 |
| 73 | g3146c | p.G1049A | 0.89 |
| 74 | a1490g | p.N497S | 0.89 |
| 75 | g1193a | p.R398H | 0.89 |
| 76 | c178a | p.Q60K | 0.88 |
| 77 | c1214t | p.S405F | 0.88 |
| 78 | g1030a | p.V344M | 0.88 |
| 79 | c3139a_a3140c | p.H1047T | 0.87 |
| 80 | g1658a | p.S553N | 0.86 |
| 81 | g3129a | p.M1043I | 0.86 |
| 82 | g3109a | p.E1037K | 0.86 |
| 83 | g3136a_a3138g | p.A1046T | 0.84 |
| 84 | WT | WT | 0.84 |
| 85 | a3154g | p.T1052A | 0.83 |
| 86 | g1357a | p.E453K | 0.83 |
| 87 | t2017a | p.S673T | 0.81 |
| 88 | a3118g | p.M1040V | 0.81 |
| 89 | c3137t | p.A1046V | 0.80 |
| 90 | a3121g | p.K1041E | 0.80 |
| 91 | g1573a | p.E525K | 0.80 |
| 92 | a227g | p.E76G | 0.80 |
| 93 | t1568c | p.L523S | 0.80 |
| 94 | c1371g | p.N457K | 0.80 |
| 95 | g238a | p.E80K | 0.80 |
| 96 | g1807c | p.D603H | 0.79 |
| 97 | g323a | p.R108H | 0.79 |
| 98 | c3104t | p.A1035V | 0.77 |
| 99 | g1133t | p.C378F | 0.77 |
| 100 | t1492a | p.W498R | 0.76 |
| 101 | a656g | p.Q219R | 0.75 |
| 102 | WT | WT | 0.74 |
| 103 | GFP | GFP | 0.74 |
| 104 | t1592c | p.L531P | 0.74 |
| 105 | g3019c | p.G1007R | 0.73 |
| 106 | g3034a | p.E1012K | 0.73 |
| 107 | g3012a | p.M1004I | 0.73 |
| 108 | a1700g | p.K567R | 0.73 |
| 109 | c3059t | p.A1020V | 0.71 |
| 110 | a1546g | p.R516G | 0.70 |
| 111 | GFP | GFP | 0.69 |
| 112 | c3074t | p.T1025I | 0.67 |
| 113 | g115a | p.E39K | 0.66 |
| 114 | a604g | p.N202D | 0.65 |
| 115 | a3184g | p.I1062V | 0.65 |
| 116 | c311t | p.P104L | 0.64 |
| 117 | t1840a | p.F614I | 0.62 |
| 118 | c1886g | p.S629C | 0.26 |

**Supplementary Table 8**

Ranking of *PIK3CA* mutations induced PI3Kα specific inhibitor BYL719 response.

| **Ranking** | **cDNA change** | **AA change** | **fold change (BYL719 D14/D14)** |
| --- | --- | --- | --- |
| 1 | GFP | GFP | 1.99 |
| 2 | GFP | GFP | 1.92 |
| 3 | c3139a_a3140c | p.H1047T | 1.57 |
| 4 | c178a | p.Q60K | 1.53 |
| 5 | a604g | p.N202D | 1.40 |
| 6 | c1598t | p.A533V | 1.30 |
| 7 | g238a | p.E80K | 1.28 |
| 8 | a227g | p.E76G | 1.26 |
| 9 | c311t | p.P104L | 1.23 |
| 10 | t1592c | p.L531P | 1.22 |
| 11 | a1490g | p.N497S | 1.21 |
| 12 | g3136a_a3138g | p.A1046T | 1.19 |
| 13 | g3034a | p.E1012K | 1.18 |
| 14 | g1357a | p.E453K | 1.16 |
| 15 | g3012a | p.M1004I | 1.15 |
| 16 | c1371g | p.N457K | 1.14 |
| 17 | t1840a | p.F614I | 1.14 |
| 18 | c1411g | p.P471A | 1.13 |
| 19 | c3137t | p.A1046V | 1.12 |
| 20 | a656g | p.Q219R | 1.11 |
| 21 | a3184g | p.I1062V | 1.10 |
| 22 | a1700g | p.K567R | 1.08 |
| 23 | t1492a | p.W498R | 1.08 |
| 24 | a153t | p.K51N | 1.07 |
| 25 | g1030a | p.V344M | 1.07 |
| 26 | a1543g | p.N515D | 1.07 |
| 27 | g2119a | p.E707K | 1.06 |
| 28 | g1639a | p.E547K | 1.05 |
| 29 | WT | WT | 1.05 |
| 30 | a3118g | p.M1040V | 1.05 |
| 31 | t1568c | p.L523S | 1.05 |
| 32 | g1624c | p.E542Q | 1.05 |
| 33 | g1807c | p.D603H | 1.04 |
| 34 | t632g | p.I211S | 1.04 |
| 35 | c1214t | p.S405F | 1.03 |
| 36 | g323a | p.R108H | 1.02 |
| 37 | g115a | p.E39K | 1.02 |
| 38 | t1395g | p.N465K | 1.02 |
| 39 | c2296t | p.L766F | 1.01 |
| 40 | g3120a | p.M1040I | 1.00 |
| 41 | g1612t | p.D538Y | 1.00 |
| 42 | c3074t | p.T1025I | 1.00 |
| 43 | g1573a | p.E525K | 0.98 |
| 44 | c1636g | p.Q546E | 0.98 |
| 45 | g1658a | p.S553N | 0.98 |
| 46 | a2279t | p.Q760L | 0.98 |
| 47 | c2965g | p.L989V | 0.97 |
| 48 | g3146c | p.G1049A | 0.97 |
| 49 | a3154g | p.T1052A | 0.96 |
| 50 | a2129g | p.E710G | 0.96 |
| 51 | a1173g | p.I391M | 0.95 |
| 52 | c3104t | p.A1035V | 0.95 |
| 53 | c3059t | p.A1020V | 0.95 |
| 54 | a1033c | p.N345H | 0.95 |
| 55 | g328a | p.E110K | 0.95 |
| 56 | a3143t | p.H1048L | 0.94 |
| 57 | g1633c | p.E545Q | 0.94 |
| 58 | a1049g | p.D350G | 0.94 |
| 59 | g1193a | p.R398H | 0.94 |
| 60 | a3121g | p.K1041E | 0.94 |
| 61 | a93g | p.I31M | 0.93 |
| 62 | a3130t | p.N1044Y | 0.92 |
| 63 | a1474g | p.I492V | 0.92 |
| 64 | c2155g | p.L719V | 0.91 |
| 65 | g333t | p.K111N | 0.91 |
| 66 | a2816g | p.D939G | 0.91 |
| 67 | a1546g | p.R516G | 0.91 |
| 68 | g263a | p.R88Q | 0.90 |
| 69 | WT | WT | 0.90 |
| 70 | g241a | p.E81K | 0.88 |
| 71 | g3129a | p.M1043I | 0.87 |
| 72 | g353a | p.G118D | 0.87 |
| 73 | c1097g | p.P366R | 0.86 |
| 74 | g113a | p.R38H | 0.86 |
| 75 | a88g | p.M30V | 0.86 |
| 76 | a1034t | p.N345I | 0.85 |
| 77 | g3085c | p.D1029H | 0.85 |
| 78 | g2176a | p.E726K | 0.83 |
| 79 | c2727a | p.F909L | 0.83 |
| 80 | g3109a | p.E1037K | 0.83 |
| 81 | t2017a | p.S673T | 0.81 |
| 82 | a1625t | p.E542V | 0.80 |
| 83 | a3127g | p.M1043V | 0.80 |
| 84 | a1625g | p.E542G | 0.79 |
| 85 | g1093a | p.E365K | 0.79 |
| 86 | g2908a | p.E970K | 0.79 |
| 87 | c1616g | p.P539R | 0.77 |
| 88 | a1094t | p.E365V | 0.77 |
| 89 | c1618t | p.L540F | 0.76 |
| 90 | g1252a | p.E418K | 0.76 |
| 91 | g1624a | p.E542K | 0.74 |
| 92 | g1633a | p.E545K | 0.73 |
| 93 | c3074g | p.T1025S | 0.73 |
| 94 | a3194t | p.H1065L | 0.72 |
| 95 | a3062g | p.Y1021C | 0.72 |
| 96 | t3132g | p.N1044K | 0.72 |
| 97 | a3143g | p.H1048R | 0.70 |
| 98 | c3197t | p.A1066V | 0.70 |
| 99 | g2740a | p.G914R | 0.70 |
| 100 | c3139t | p.H1047Y | 0.68 |
| 101 | a1637g | p.Q546R | 0.68 |
| 102 | c3155a | p.T1052K | 0.67 |
| 103 | t1035g | p.N345K | 0.66 |
| 104 | a1637t | p.Q546L | 0.66 |
| 105 | g1624a_a1625g | p.E542R | 0.63 |
| 106 | t1258c | p.C420R | 0.61 |
| 107 | g1635t | p.E545D | 0.60 |
| 108 | g3019c | p.G1007R | 0.59 |
| 109 | a1637c | p.Q546P | 0.58 |
| 110 | g1133t | p.C378F | 0.52 |
| 111 | c1697t | p.P566L | 0.50 |
| 112 | a1634c | p.E545A | 0.49 |
| 113 | a1634g | p.E545G | 0.46 |
| 114 | g3145c | p.G1049R | 0.45 |
| 115 | g2702t | p.C901F | 0.43 |
| 116 | a3140t | p.H1047L | 0.43 |
| 117 | a3140g | p.H1047R | 0.33 |
| 118 | c1886g | p.S629C | 0.13 |

**Supplementary Table 9**

The forward and reverse primer sequences for detection of *EGFR, ERBB2, ERBB3, ERBB4, FGFR1, FGFR2, FGFR3, FGFR4, IGF-IR* by qPCR.

| *EGFR* | F: AGGCACGAGTAACAAGCTCAC |
| --- | --- |
|  | R: ATGAGGACATAACCAGCCACC |
| *ERBB2* | F: TGCAGGGAAACCTGGAACTC |
|  | R: ACAGGGGTGGTATTGTTCAGC |
| *ERBB3* | F: GGTGATGGGGAACCTTGAGAT |
|  | R: CTGTCACTTCTCGAATCCACTG |
| *ERBB4* | F: GTCCAGCCCAGCGATTCTC |
|  | R: AGAGCCACTAACACGTAGCCT |
| *FGFR1* | F: CCCGTAGCTCCATATTGGACA |
|  | R: TTTGCCATTTTTCAACCAGCG |
| *FGFR2* | F: AGCACCATACTGGACCAACAC |
|  | R: GGCAGCGAAACTTGACAGTG |
| *FGFR3* | F: TGCGTCGTGGAGAACAAGTTT |
|  | R: GCACGGTAACGTAGGGTGTG |
| *FGFR4* | F: GAGGGGCCGCCTAGAGATT |
|  | R: CAGGACGATCATGGAGCCT |
| *IGF-1R* | F: TCGACATCCGCAACGACTATC |
|  | R: CCAGGGCGTAGTTGTAGAAGAG |
